# Radical 1,2-boron shift in modern organoboron synthesis

**DOI:** 10.3389/fchem.2026.1955064

**Published:** 2026-09-28

**Authors:** Yunkai Zhang, Jinduo Shen, Qiuren Wei, Xi Li, Zhongyan Cao, Kai Sun, Lingbo Qu, Bing Yu

**Affiliations:** 1 College of Chemistry, Zhengzhou University, Zhengzhou, China; 2 Hunan Provincial University Key Laboratory of the Fundamental and Clinical Research on Functional Nucleic Acid, Changsha Medical University, Changsha, China; 3 College of Chemistry and Molecular Sciences, Henan University, Kaifeng, China; 4 International Innovation Center for Forest Chemicals and Materials, Nanjing Forestry University, Nanjing, China

**Keywords:** organoboron synthesis, photocatalysis, radical 1,2-boron shift, skeletal editing, β-boryl radical

## Abstract

The radical-mediated 1,2-boron shift strategy has emerged as a promising and powerful approach for the construction of functionalized organoboron compounds. This review summarizes and categorizes the relevant studies based on the distinct mechanisms underlying the generation of β-boryl radicals, such as radical addition, oxidative cleavage, decarboxylation reactions, or hydrogen atom transfer (HAT). Furthermore, the reaction scopes, synthetic utilities, and underlying mechanistic pathways are thoroughly examined to provide a comprehensive overview of this evolving strategy.

## 1 Introduction

Organoboron compounds play a central role in organic synthesis and pharmaceutical chemistry, owing to their distinctive electronic structures and reactivity profiles (Lennox and Lloyd-Jones, 2014; Lu et al., 2021; Miyaura and Suzuki, 1995; Xu et al., 2018). The seminal discovery of the Suzuki-Miyaura coupling reaction in 1979 marked a pivotal advancement in organoboron chemistry (Lennox and Lloyd-Jones, 2014), facilitating the formation of C-C bonds by coupling aryl halides with arylboronic acids in the presence of palladium (Pd) catalysts and bases (Figure 1a; Das and Linert, 2016). Since its inception, the Suzuki-Miyaura coupling has emerged as one of the most widely utilized methods for C-C bond formation (Hu and Kazemi, 2024; Liu et al., 2014a; Liu et al., 2014b; Liu et al., 2014c; Liu et al., 2018; Liu et al., 2019; Xu and Duan, 2019), underscoring its significance in the synthesis of complex organic molecules.

**FIGURE 1:**
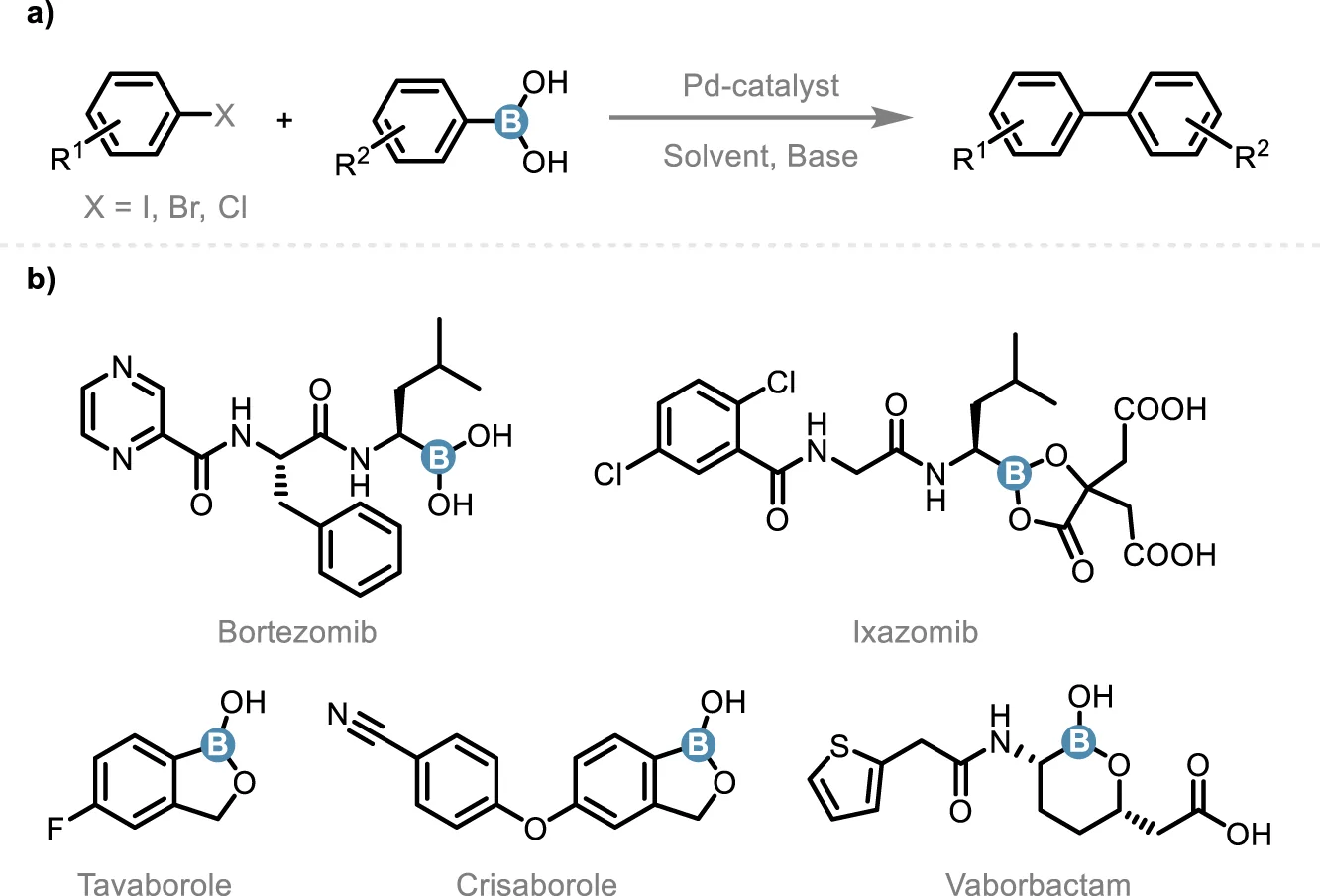
The importance of organoboron compounds. **(a)** Suzuki–Miyaura cross-coupling reactions, **(b)** FDA-approved boron-containing drugs.

In addition to its synthetic utility, the role of boron in drug design has gained increasing prominence, becoming a critical focus in medicinal chemistry (Wang and Liu; Dembitsky et al., 2011; Smoum et al., 2012; Li et al., 2025). Boron-containing compounds have proven to be indispensable in targeting novel therapeutic sites, offering potential for the development of innovative treatments for various diseases (Hu et al., 2024; Wang et al., 2024; Xiong et al., 2025). Noteworthy examples of boron-based therapeutics include Bortezomib, Ixazomib, Tavaborole, Crisaborole, and Vaborbactam (Figure 1b), each illustrating the capability of organoboron chemistry to address complex medical challenges (Messner et al., 2022). These agents highlight the evolving therapeutic potential of organoboron compounds, which continue to provide novel mechanisms of action for treatment (Fyfe and Watson, 2017). As the field of organoboron chemistry continues to progress, the synthesis and derivatization of organoboron compounds remain increasingly vital (Song et al., 2021).

More recently, driven by advances in radical chemistry, radical-mediated 1,2-boron shift have emerged as a powerful and conceptually novel synthetic tool (McCallum, 2023). As early as 1999, Batey and Smil reported the first example of a 1,2-boron shift occurring during a radical cyclization (Batey and Smil, 1999). Unlike traditional ionic pathways that typically commence with the formation of a tetracoordinate boronate (ate) complex and often require strongly activating conditions (Shen et al., 2026), the radical-driven approach offers distinct advantages: it operates under mild conditions, effectively overcomes steric constraints to construct complex quaternary carbon centers, and exhibits excellent functional group tolerance (Kaiser et al., 2019).

Over the past 2 decades, the 1,2-boron shift strategy has attracted considerable attention for the construction of complex organoboron compounds. The resulting organoboron products not only represent valuable structural motifs themselves, but their intact C–B bonds are amenable to further transformations, greatly enhancing their utility in assembling polyfunctionalized molecules (Xu et al., 2015). While ionic 1,2-boron shift have been extensively studied, investigations into radical-mediated variants remain comparatively limited (Jiang et al., 2022). This emerging radical 1,2-boron shift strategy provides a new avenue to overcome the limitations of traditional organoboron chemistry, enabling the efficient synthesis of highly valuable and structurally complex organoboron pharmaceutical molecules. The key to achieving such radical-type 1,2-boron shift lies in the generation of the crucial β-boryl radical intermediate, which subsequently undergoes the 1,2-boron shift driven by a favorable thermodynamic driving force. DFT computations indicate that boron migration converts the primary radical into a secondary alkyl radical via a transition state with an energy barrier of only 8.1 kcal·mol^-1^, yielding a product that is 4.5 kcal·mol^-1^ more stable than the initial state (Figure 2a; Kaiser et al., 2019).

**FIGURE 2:**
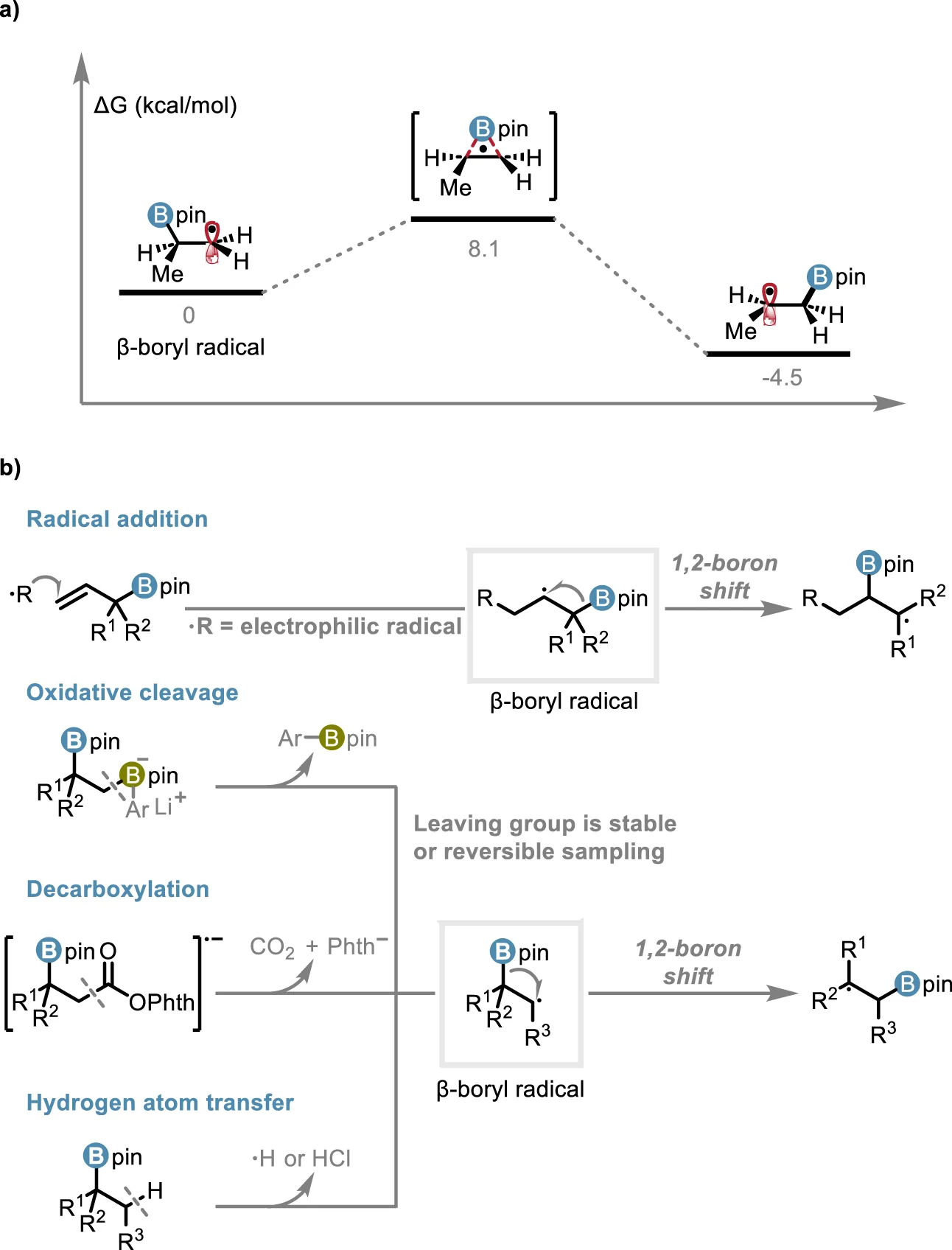
Radical 1,2-boron shift: thermodynamic factors and β-boryl radical generation strategies. **(a)** Thermodynamic factors of radical induced 1,2-boron shift, **(b)** Strategies for constructing β-boryl radical.

To generate these crucial β-boryl radicals, diverse activation modes have been developed. Photocatalysis has become the prevailing and widely adopted approach for triggering 1,2-boron shift owing to its mild and sustainable characteristics (Cui et al., 2025; Liu et al., 2020; Qiao et al., 2026; Song et al., 2026; Sun et al., 2021; Sun et al., 2024; Wang et al., 2025; Wang et al., 2026; Yang et al., 2025; Yang et al., 2026; Zeng et al., 2026; Zhang Z. et al., 2026). Meanwhile, thermal initiation, albeit less frequently documented, serves as an indispensable and valuable complementary activation mode. While previous excellent reviews by Gao (Jiang et al., 2022) and McCallum. (2023) have covered aspects of 1,2-boron shift, a comprehensive and up-to-date summary focusing specifically on photo- and thermo-driven 1,2-boron shift remains absent. In light of this gap, the aim of this review is to consolidate the latest advancements in the construction of functional organoboron compounds through photocatalysis or thermal initiation-mediated 1,2-boron shift. It is worth noting that the radical 1,2-boron shift discussed in this review specifically refers to the migration of a boryl group between two adjacent carbon atoms. Other related types of boron migration, such as the migration of a boryl group from a carbon atom to an adjacent oxygen atom, fall outside the scope of this review (Kumar et al., 2025). Consequently, this review summarizes and categorizes the relevant research based on the diverse mechanisms for generating β-boryl radicals, such as radical addition, oxidative cleavage, decarboxylation, or hydrogen atom transfer (HAT) (Figure 2b). We firmly believe that the radical-mediated 1,2-boron shift strategy serves as a transformative tool in organoboron synthesis, and this review will inspire chemists to design increasingly sophisticated and impactful molecular architectures.

## 2 1,2-Bis-boronic esters as precursors

1,2-Bis-boronic esters represent a class of pivotal building blocks, readily accessible via the diboration of alkenes (Bonet et al., 2011). The two boronic groups within these molecules exhibit distinct reactivities owing to differences in steric environments, providing a unique platform for the construction of complex molecules through sequential, site-selective functionalization (Wang et al., 2022a), as the transmetalation step in conventional metal-catalyzed cross-couplings is exquisitely sensitive to steric hindrance around the boron center. However, dictated by steric effects, traditional transformations have remained predominantly restricted to the less hindered primary carbon-boron (C-B) sites (Wang et al., 2022a). Overcoming this deep-seated regioselectivity to achieve precise mono-functionalization of the more challenging and sterically congested secondary or tertiary boronic sites remains a long-standing challenge for synthetic chemists (Wang et al., 2022b).

In 2019, the Aggarwal group ingeniously integrated photoredox catalysis to develop a novel indirect activation mode (Kaiser et al., 2019). This strategy utilizes 1,2-bis-boronic esters and electron-deficient alkenes as starting materials. Under activation by an aryllithium reagent, the reaction is driven by the organic photocatalyst 4CzIPN under blue-light irradiation. A series of 1,2-bis-boronic esters featuring diverse steric hindrances and functional group substitutions can react smoothly with various electron-deficient alkenes, achieving Giese-type additions with yields ranging from 35% to 98% (Scheme 1). More remarkably, this strategy demonstrates robust generality and synthetic potential: when applied to 1,2,3-triboronic esters, two consecutive 1,2-boron shift can occur to efficiently construct stable tertiary carbon centers with high selectivity, corroborating the thermodynamically controlled nature of the reaction. Furthermore, a transannular radical cyclization achieved using cyclooctadiene derivatives allows for the single-step construction of challenging bicyclic frameworks, highlighting its value for assembling complex molecules.

**SCHEME 1:**
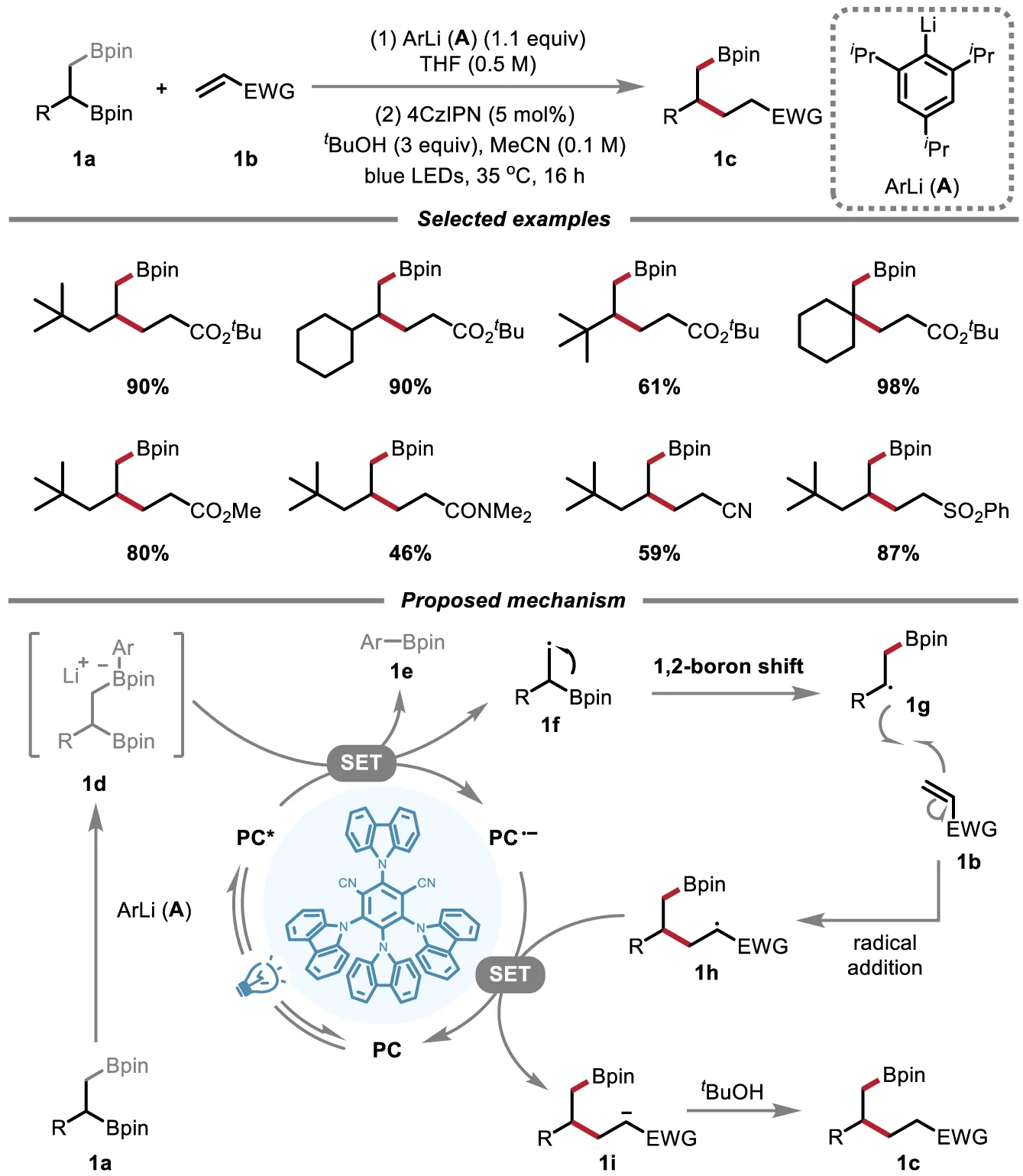
Photocatalytic secondary-selective functionalization of 1,2-bis-boronic esters.

Mechanistic studies indicate that the visible-light-excited photocatalyst (PC*) oxidizes the activated boronic ester **1d** via single-electron transfer (SET), triggering the homolytic cleavage of the primary carbon-boron bond to generate the primary alkyl radical **1f** and release the arylboronic ester **1e**. This primary radical subsequently undergoes a crucial 1,2-boron shift, converting into the more stable secondary alkyl radical **1g**, which then undergoes a radical addition with the electron-deficient alkene **1b**. Ultimately, the addition intermediate acquires an electron from the reduced catalyst and undergoes protonation to afford the final product **1c**, simultaneously completing the catalytic cycle.

While the former protocol successfully validated the 1,2-boron shift strategy for site-selective Giese-type alkylations, extending this transformation to forge synthetically privileged C(sp^3^)–C(sp^2^) bonds (particularly arylation reactions) represented the next critical objective (Fyfe and Watson, 2017). In 2022, the Aggarwal group reported an innovative photochemical arylation mode, the core of which lies in the utilization of electron donor-acceptor (EDA) complexes to achieve transformations without the need for an external photocatalyst (Wang et al., 2022b). This strategy employs 1,2-bis-boronic esters and (hetero)aryl nitriles as starting materials. Through the activation of the boronic ester by an electron-rich aryllithium reagent, an EDA complex is formed *in situ* with the electron-deficient aryl nitrile; the reaction is subsequently driven by blue LED irradiation under mild conditions. This method demonstrates broad generality, being applicable to a range of primary, secondary, and tertiary boronic esters, affording the target products in 32%–90% yields (Scheme 2). Its prominent value is highlighted by its stereoselective control and capacity for complex molecule construction: for cyclic *cis*-1,2-bis-boronic esters, this photochemical pathway exclusively generates *trans*-coupled products. This serves as a stereochemical complement to the *cis*-products obtained via traditional palladium-catalyzed Suzuki-Miyaura reactions, providing a novel avenue for stereodivergent synthesis. Furthermore, through a cleverly designed radical cascade, this method enables the single-step construction of complex bicyclic frameworks containing quaternary carbon centers, underscoring its value for assembling complex molecules.

**SCHEME 2:**
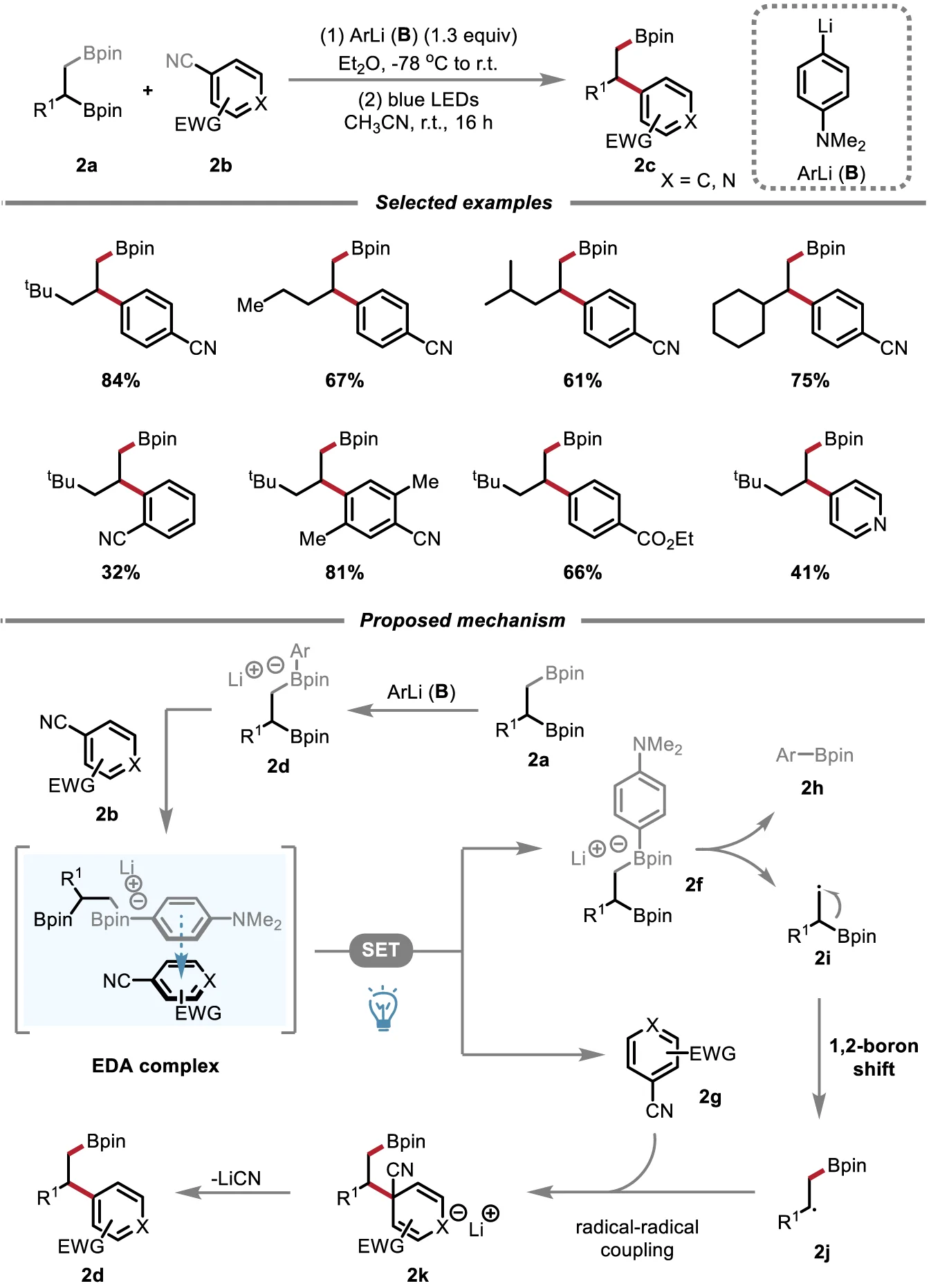
EDA complex-driven secondary-selective arylation of 1,2-bis-boronic esters.

Mechanistic studies indicate that visible light excitation of the EDA complex induces a SET from the activated boronic ester to the aryl nitrile, generating the radical ion pair **2f**. Subsequently, the homolytic cleavage of the primary carbon-boron bond releases the arylboronic ester **2h** and generates the primary β-boryl radical **2i**. This radical rapidly undergoes a crucial 1,2-boron shift, converting into the more stable secondary alkyl radical **2j**. Finally, this transient radical undergoes cross-coupling with the persistent radical anion **2g** (derived from the aryl nitrile), which, following cyanide elimination, yields the final arylated product.

While this EDA-driven protocol enables metal-free arylation with stereochemical complementarity to traditional Suzuki-Miyaura coupling, its heavy reliance on electron-deficient (hetero)aryl nitriles to stabilize key radical anion intermediates severely limits the scope of compatible aryl coupling partners. To break through this bottleneck and establish a more general catalytic platform compatible with widely available (hetero)aryl halides, in 2022, the Aggarwal group constructed a dual photoredox/nickel catalytic platform, successfully achieving the regioselective cross-coupling of 1,2-bis-boronic esters with a broad range of aryl halides (Wang et al., 2022a). This strategy integrates the organic photocatalyst 4CzIPN with a nickel catalyst to efficiently drive the reaction under mild room-temperature conditions and blue-light irradiation. The method demonstrates exceptional generality and utility: a variety of aryl bromides/iodides and differently substituted 1,2-bis-boronic esters are well-compatible, affording the target products in yields of 31%–92% (Scheme 3). Its scale-up potential was subsequently demonstrated in a gram-scale (2 mmol) experiment. Most importantly, the boronic ester group retained in the product serves as a versatile handle, allowing for the efficient preparation of high-value building blocks such as alkenes, amines, and alcohols through diverse transformations including Zweifel olefination and oxidative amination, highlighting its value for flexible molecular scaffold derivatization. To demonstrate the practical utility of this strategy, the resulting products were further derivatized.

**SCHEME 3:**
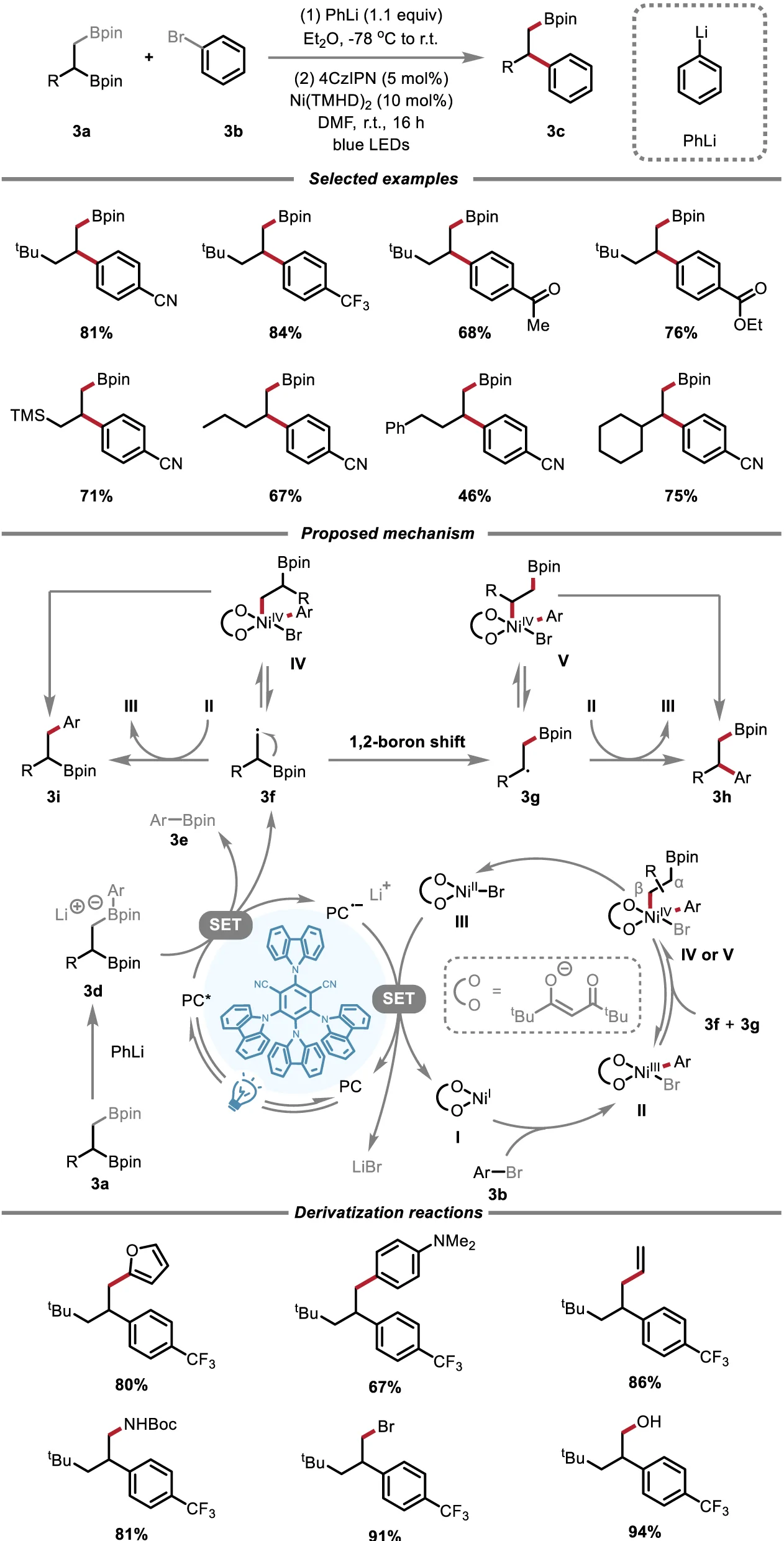
Dual nickel/photoredox-catalyzed secondary-selective arylation of 1,2-bis-boronic esters.

Mechanistic studies indicate that the activated **3d** undergoes oxidative C-B bond cleavage via an SET process mediated by the visible-light-excited photocatalyst, generating the primary β-boryl radical **3f**. This radical can either undergo a crucial 1,2-boron shift to produce the lower-energy secondary radical **3g**, or be directly intercepted by the nickel catalyst. The pathway is dictated by a competition between the rate of 1,2-boron shift and the rate of radical capture by the nickel catalyst: sterically bulky intermediates preferentially follow the shift path to yield products with tertiary carbon centers, whereas less hindered intermediates undergo direct coupling, enabling precise control over different regioselective products. Subsequently, coupling of migrated radicals is completed through classical nickel-catalyzed steps, including oxidative addition, outer-sphere reductive elimination, and catalyst regeneration.

## 3 Allylboronic esters as precursors

### 3.1 1,3-Difunctionalization reaction

Although the 1,2-difunctionalization of alkenes has reached maturity, radical-mediated 1,3-difunctionalization has long been constrained by the necessity of ring-opening in highly strained rings. Furthermore, traditional allylation reactions are frequently accompanied by the loss of high-value boryl groups, significantly limiting their synthetic applications. To break through this bottleneck, in 2019, the Aggarwal group reported a visible-light-induced hydrotrifluoromethylation strategy for allylboronic esters (Kaiser et al., 2019). This work ingeniously leverages the 1,2-boron shift mechanism to achieve highly efficient transformations under mild conditions, driven by the synergistic action of the photocatalyst 4CzIPN, sodium trifluoromethanesulfinate, and thiophenol (Scheme 4). The core breakthrough of this strategy lies in the successful retention of the boryl group within the molecule, which not only overcomes the inherent flaws of traditional methods but also provides great convenience for subsequent derivatizations of the molecular scaffold.

**SCHEME 4:**
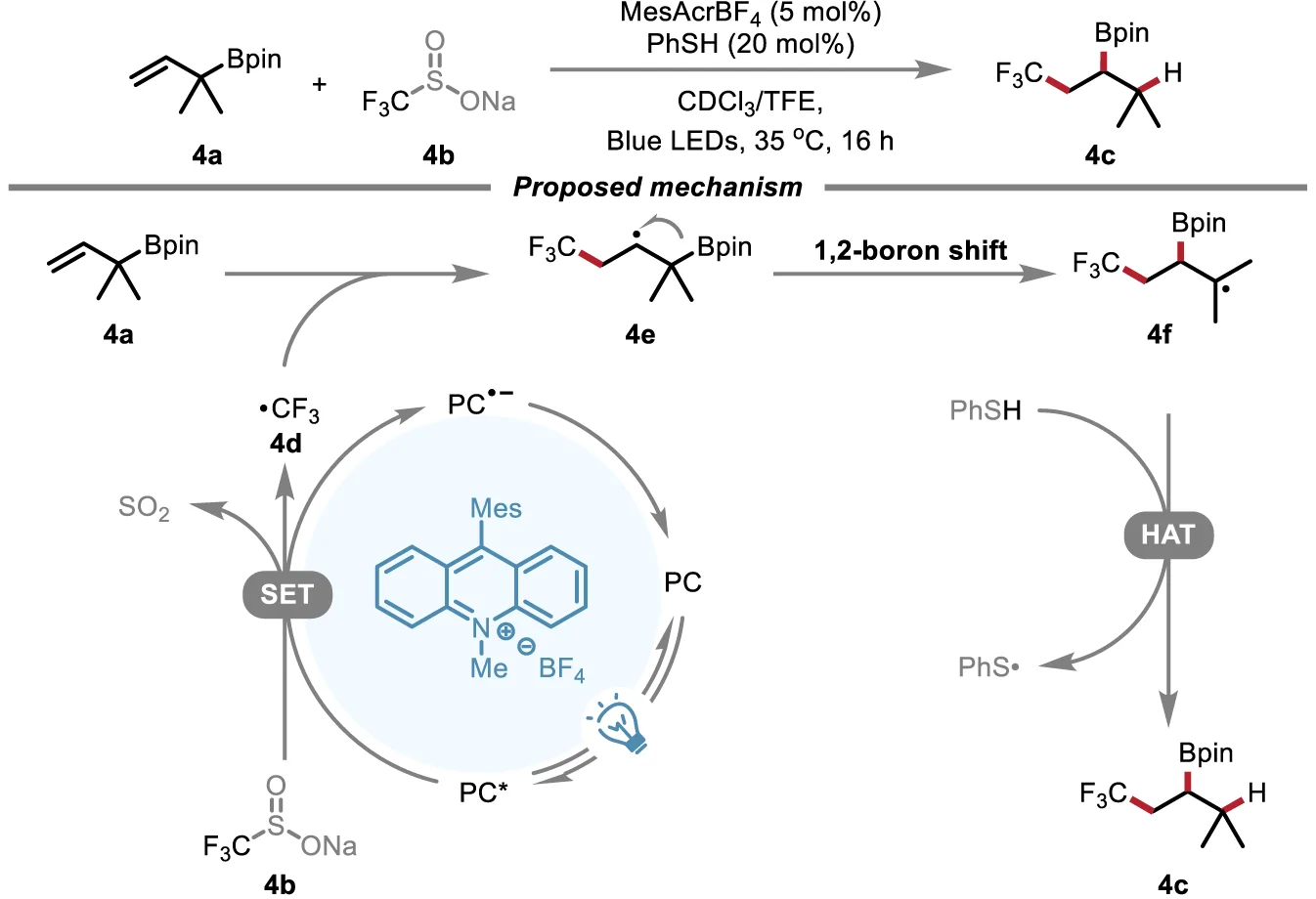
1,3-Hydrotrifluoromethylation of allylboronic esters.

Mechanistic studies indicate that the visible-light-excited catalyst releases the crucial trifluoromethyl radical **4d** via a SET process. The addition of this radical to the alkene triggers a subsequent 1,2-boron shift, generating the thermodynamically favoured intermediate **4f**, which ultimately yields the target product **4c** through a HAT process.

Applying the 1,2-boron shift strategy to the remote 1,3-difunctionalization of alkenes is a key direction for expanding its synthetic potential. In 2020, the Studer group reported a novel radical 1,3-trifluoromethylalkynylation method based on a bifunctional reagent, alkynyl trifluoromethyl sulfone (Jana et al., 2020). This reaction proceeds under AIBN initiation and heating conditions, efficiently constructing a series of 1,3-trifluoromethylalkynylated alkanes in 26%–87% yields, with the high-value boronic ester group in the molecule remaining intact (Scheme 5). The synthetic utility of this method is fully demonstrated through its diverse derivatizations. The boryl group in the product serves not only as a crucial handle for subsequent transformations, enabling the construction of high-value-added frameworks such as dihydrofurans; more importantly, this core strategy possesses excellent platform extensibility. It can be further expanded to 1,3-azidotrifluoromethylation, three-component alkylation, and allylation reactions, exhibiting broad generality.

**SCHEME 5:**
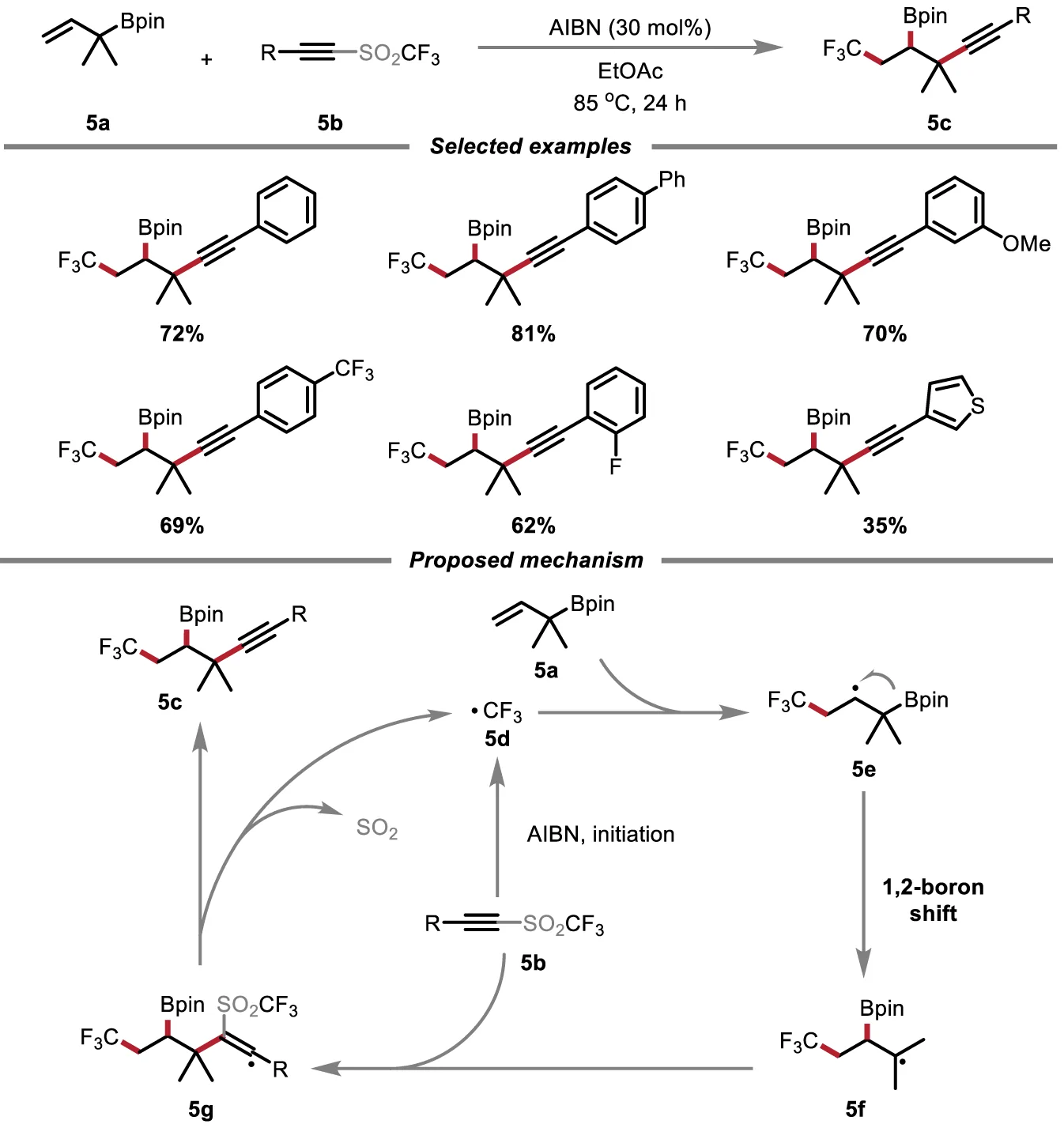
1,3-Trifluoromethylalkynylation of allylboronic esters.

Mechanistic studies indicate that the trifluoromethyl radical **5d**, generated via AIBN initiation, selectively adds to the terminal position of the alkene **5a**, triggering a crucial 1,2-boron shift to generate the more stable rearranged radical **5e**. Subsequently, this intermediate **5e** is captured by another molecule of the bifunctional reagent **5b**, which, upon β-fragmentation, introduces the alkynyl group while simultaneously regenerating the CF_3_ radical **5d**, thereby sustaining the radical chain cycle.

Further expanding the 1,2-boron shift strategy to more challenging three-component systems, the Studer group reported a novel 1,3-carboheteroarylation method for allylboronic esters in 2022 (Jana and Studer, 2022). Using allylboronic esters, quinoxalin-2(1H)-ones, and perfluoroalkyl iodides as starting materials, the reaction operates efficiently at room temperature under the synergistic promotion of DBU and blue LED irradiation, constructing a series of complex multisubstituted molecules in 46%–85% yields (Scheme 6). The synthetic advantages of this system are also reflected in its excellent practicality and capability for functional group retention. The reaction can not only be easily scaled up to a 1 mmol scale, but the high-value boronic ester group in the product is also preserved. This group can serve as a useful synthetic handle directly for subsequent transformations such as oxidation or cross-coupling, demonstrating its immense potential in the scalable synthesis of complex molecules such as pharmaceutical intermediates.

**SCHEME 6:**
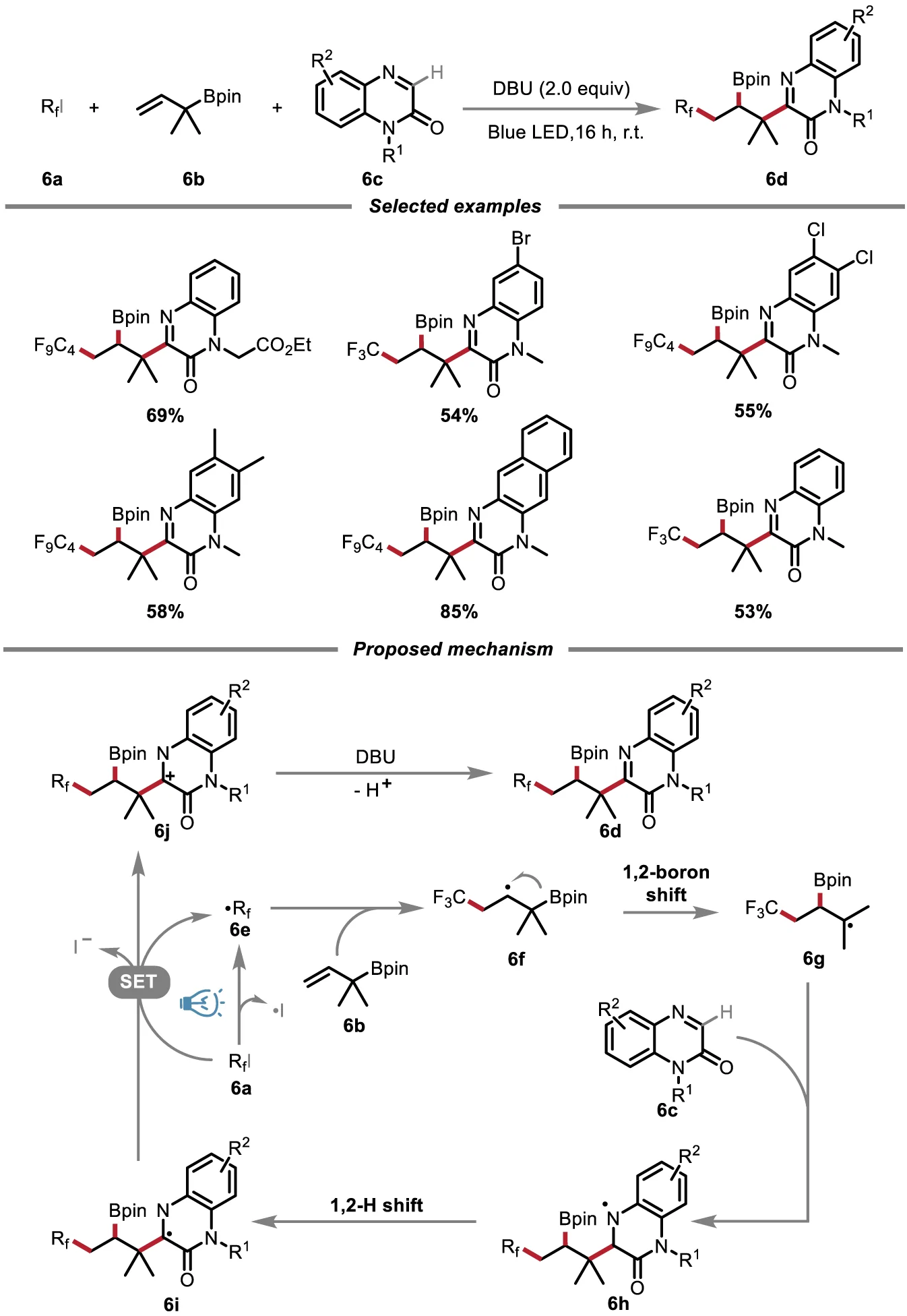
1,3-Carboheteroarylation of allylboronic esters.

Mechanistic studies indicate that light irradiation initiates the homolytic cleavage of the perfluoroalkyl iodide to generate radical **6e** and an iodine radical. Radical **6e** regioselectively adds to the terminal position of alkene **6b** and triggers a crucial 1,2-boron shift to generate the lower-energy rearranged radical **6g**. This intermediate subsequently undergoes a heteroarylation addition to **6c**, and following a 1,2-hydrogen shift and oxidative deprotonation, yields the final product **6d** while simultaneously regenerating radical **6e** to maintain the chain reaction cycle.

In the same work, the Studer group further extended the 1,2-boron shift strategy to the 1,3-hydroalkylation of allylboronic esters (Jana and Studer, 2022). This system utilizes Michael acceptors as alkylating reagents and, under the synergistic action of iron catalysis and a hydrosilane reduction system, operates efficiently under mild heating conditions to construct a series of 1,3-difunctionalized products in 38%–71% yields (Scheme 7). Notably, for specific substrates, this reaction exhibits excellent regio- and diastereoselectivity, opening a new avenue for the stereoselective synthesis of chiral boronic ester building blocks.

**SCHEME 7:**
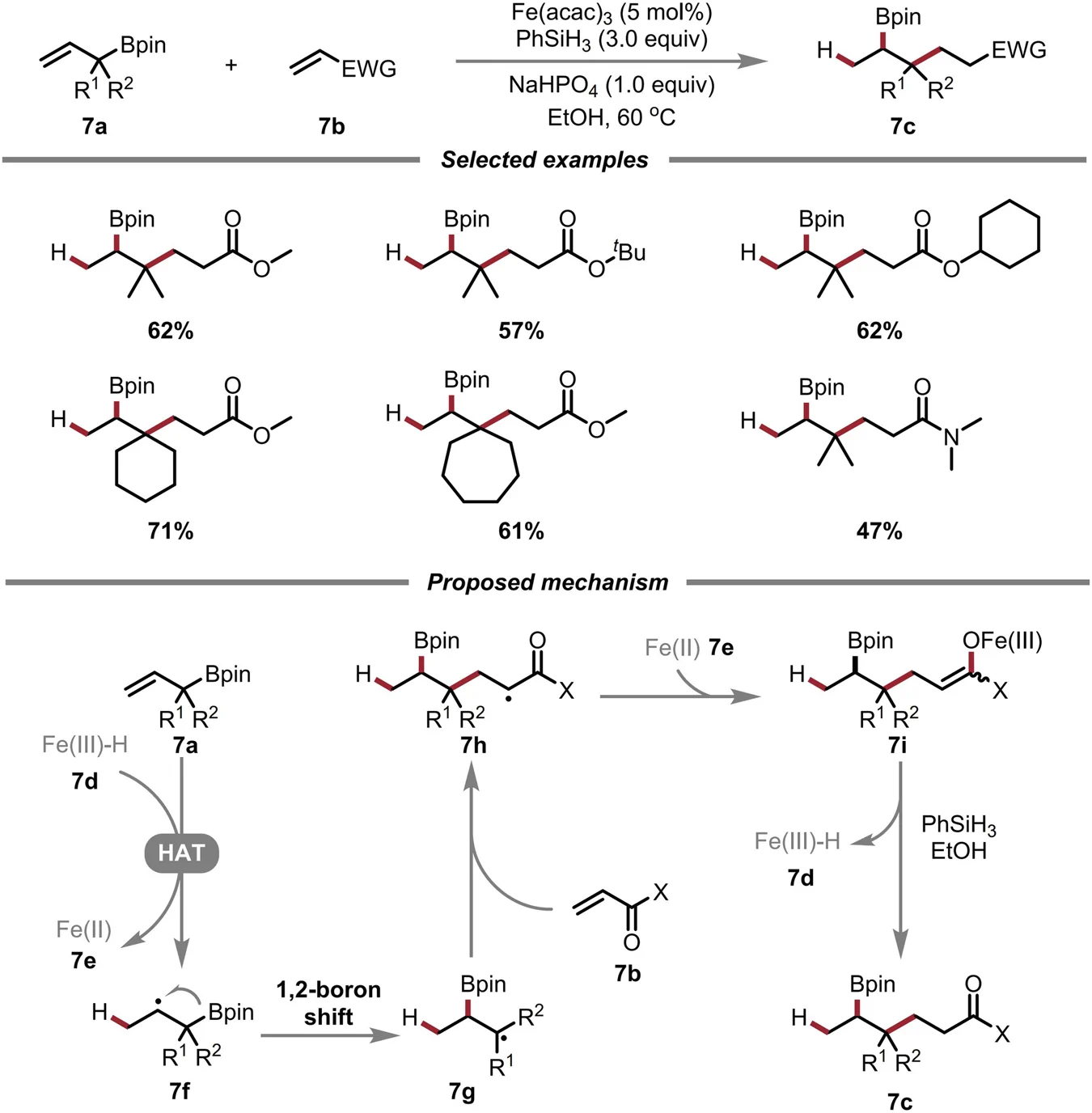
1,3-Hydroalkylation of allylboronic esters.

Mechanistically, the reaction is initiated by an Fe(III)-H species generated *in situ* from the Fe(III) catalyst, which regioselectively adds to alkene **7a** via a HAT process, which triggers the crucial 1,2-boron shift to generate the thermodynamically favoured and nucleophilic α-boryl alkyl radical **7g**. This intermediate **7g** subsequently undergoes efficient coupling with the electrophilic Michael acceptor **7b**. The resulting adduct radical is then captured by Fe(II) and undergoes reductive protonation to release the product **7c** and regenerate the active Fe(III)-H species, smoothly completing the catalytic cycle.

In 2025, the Yu and Chen group developed a novel visible-light-induced 1,3-difunctionalization strategy for allylboronic esters, successfully achieving the precise construction of *gem*-difluoroalkenes (Xiang et al., 2025). This system utilizes an iridium complex as the photocatalyst to drive the cascade transformation of allylboronic esters and vinyl trifluoromethanesulfonates under mild light irradiation conditions, constructing a series of structurally diverse target molecules in excellent yields ranging from 22% to 95% (Scheme 8). This strategy not only exhibits remarkable substrate generality and late-stage derivatization capability, but also strongly demonstrates its high practical utility in drug discovery and industrial applications through a simplified one-pot synthetic protocol, successful gram-scale scale-up, and broad compatibility with sulfonylating reagents.

**SCHEME 8:**
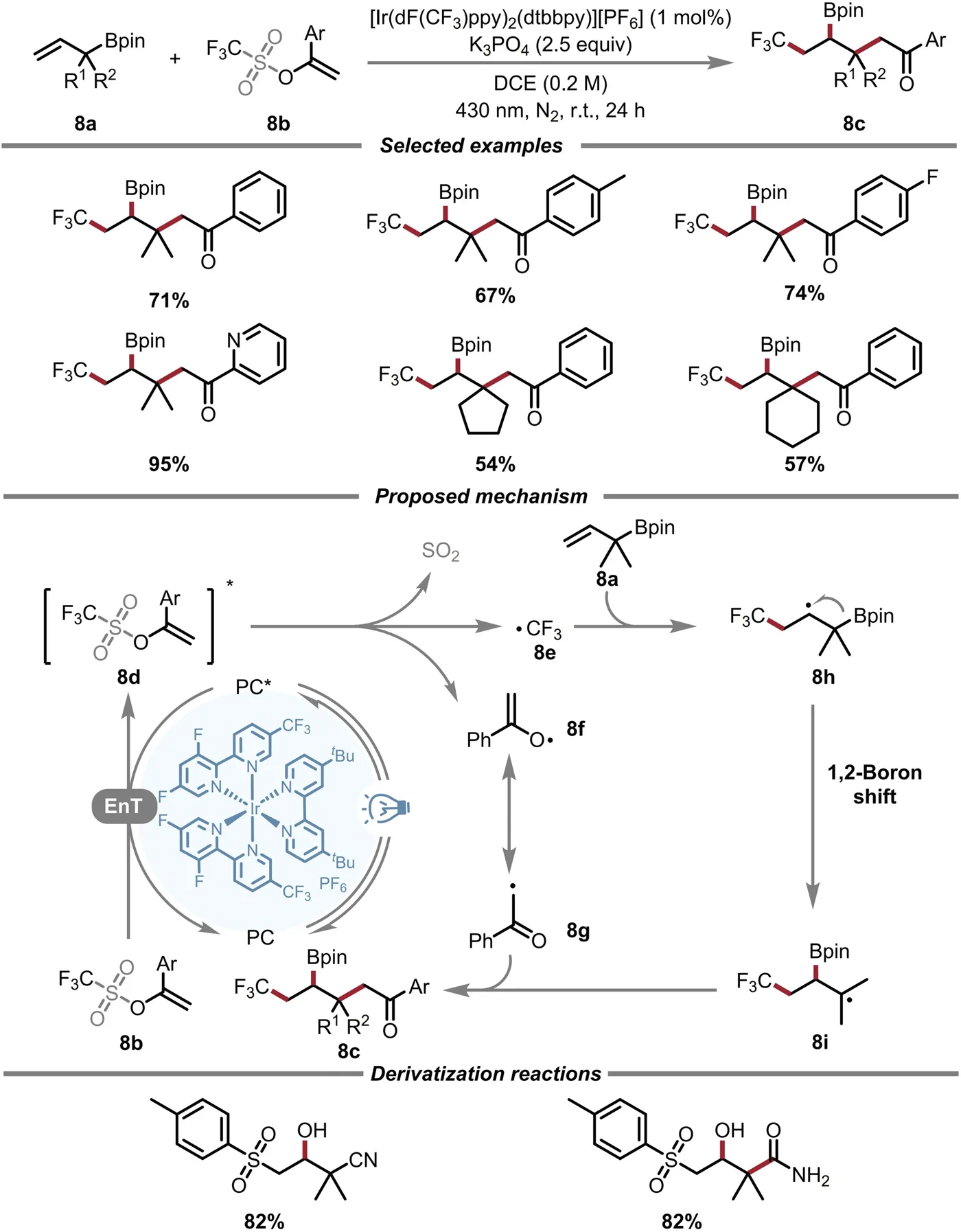
1,3-Trifluoromethylcarbonylation of allylboronic esters.

Mechanistic studies indicate that the light-excited catalyst induces the homolytic cleavage of the trifluoromethanesulfonate via triplet energy transfer, releasing the trifluoromethyl radical **8e**, which then adds to the allylboronic ester **8a**. This immediately triggers a thermodynamically driven 1,2-boron shift, reshaping it into the more stable carbon-centered radical **8i**. Meanwhile, the *in situ* generated α-carbonyl radical **8g** undergoes highly efficient radical-radical cross-coupling with the migrated radical intermediate **8i**, precisely constructing the 1,3-difunctionalized framework while simultaneously regenerating the catalyst to complete the catalytic cycle.

### 3.2 1,3-Remote boron shift

While 1,2-boron shift has emerged as a precise strategy for 1,3-difunctionalization and witnessed significant advances, achieving more remote boron positioning remains challenging. Direct 1,3-boron migration has long suffered from a formidable kinetic barrier due to the highly strained four-membered-ring transition state involved. To address this longstanding issue, the Wang group devised an ingenious workaround in 2022, elegantly merging two consecutive 1,2-boron shift with a Smiles-type rearrangement to accomplish the net 1,3-boron migration (Tao et al., 2022). Utilizing 10-phenylphenothiazine (PTH) as the photocatalyst under blue-light irradiation, this strategy drives the cascade transformation of sodium arylsulfinates and allylboronic esters. Through an ingenious design of achieving a one-step 1,3-shift via two successive 1,2-shifts, it successfully constructs thermodynamically challenging remote boron-migrated products. This system exhibits exceptional flexibility; a diverse range of arylsulfinate and allylboronic ester substrates can undergo smooth conversion, affording the target molecules in 25%–87% yields (Scheme 9). More importantly from an application perspective, by simply tuning the reaction conditions, the same intermediate can be directed toward three distinct pathways: highly efficiently yielding the 1,3-boron shift product under standard conditions; and achieving either a dearomative cyclization or a 1,2-difunctionalization upon altering the conditions. This provides a versatile toolbox for synthesizing structurally diverse organoboron compounds.

**SCHEME 9:**
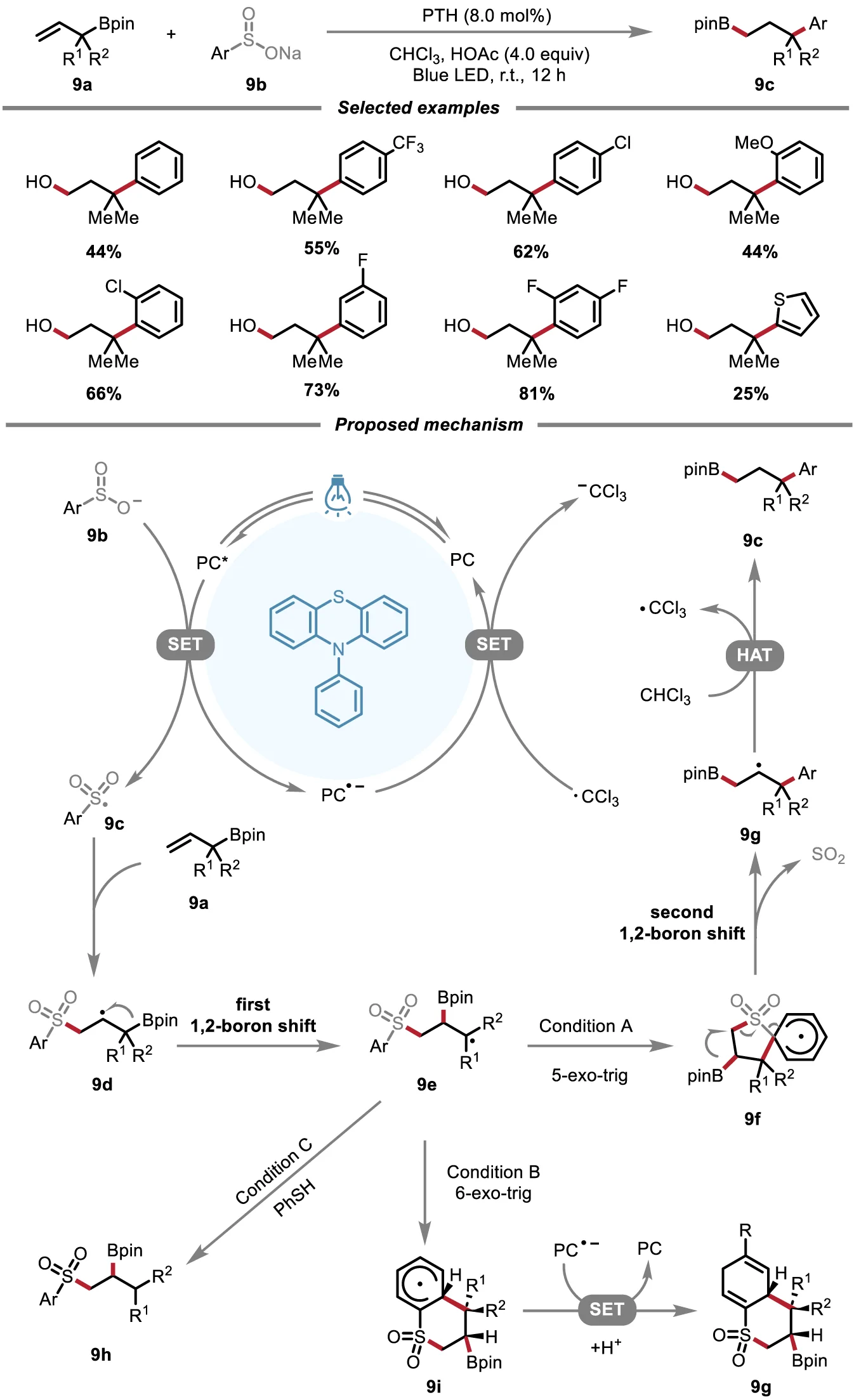
Photocatalytic successive 1,2-boron shift enabling remote 1,3-boron shift.

Mechanistic studies indicate that the light-excited PTH catalyzes the sodium arylsulfinate **9b** to generate the sulfonyl radical **9c**. This radical subsequently adds to the allylboronic ester **9a**, triggering the first 1,2-boron shift to afford the key intermediate **9e**. Under condition A, the key intermediate **9e** subsequently undergoes an intramolecular cyclization and a Smiles rearrangement, extruding SO_2_, while simultaneously initiating a second 1,2-boron shift to yield intermediate **9g**, which ultimately completes the process via HAT. Under condition B, an *exo*-cyclization occurs to afford the dearomatized product **9g**; under condition C, the intermediate is captured by a thiophenol, generating the 1,2-difunctionalized boronic ester **9h**.

### 3.3 Synthesis of functionalized borosilanes

To address the current reliance on transition metals and the lack of regiocontrol strategies in the traditional synthesis of *vic*-borosilanes, the Wu group developed a novel visible-light-driven HAT catalysis strategy in 2023, successfully achieving the γ-selective hydrosilylation of allylboronic esters (Kong et al., 2023). This system ingeniously leverages the synergistic catalysis of an organic photosensitizer and a thiol. Under mild light irradiation conditions, it overcomes the regioselectivity bottleneck of radical hydrosilylation, constructing a series of high-value *vic*-borosilane frameworks in 52%–88% yields (Scheme 10).

**SCHEME 10:**
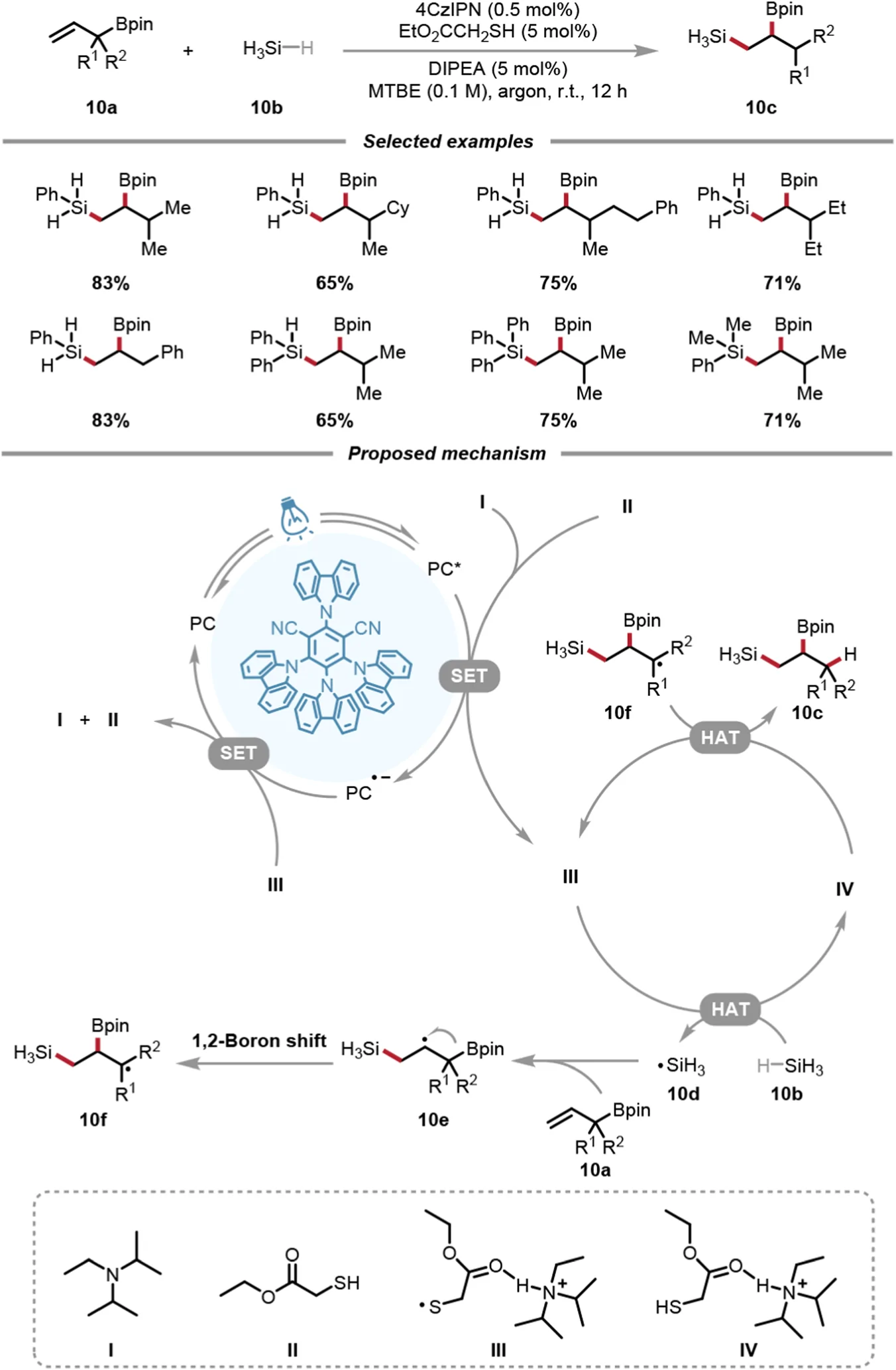
1,3-Hydrosilylation of allylboronic esters.

Insightful DFT calculations and control experiments suggest that the photo-induced thiyl radical **III** generates the nucleophilic silyl radical **10d** via hydrogen abstraction. Following the principle of polarity matching, this intermediate exclusively attacks the less sterically hindered γ-position of the allylboronic ester **10a**. This subsequently triggers a thermodynamically driven 1,2-boron shift, reshaping it into the more stable α-carbon radical intermediate **10f**. Ultimately, the final product **10c** is obtained through a hydrogen atom transfer process, simultaneously regenerating **III** to close the catalytic cycle.

### 3.4 1,2,5-Trifunctionalization reaction

Traditional radical 1,5-difunctionalization strategies are often limited by highly customized precursors and restricted functional group compatibility, making it difficult to flexibly introduce functional groups at the two-position. To break through this limitation, in 2023, the Glorius group developed a novel visible-light energy transfer catalysis-driven strategy for the three-component 1,2,5-trifunctionalization of alkenes (Paulus et al., 2023). The logical evolution of this reaction lies in utilizing the 1,2-boron shift as the starting point for a radical relay, enabling multi-site functionalization by extending the span of the carbon chain. This system ingeniously integrates an imine-based bifunctional reagent, an allylboronic ester, and a Michael acceptor. Under the action of a catalytic amount of an [Ir-F] catalyst, the reaction operates efficiently in ethyl acetate under blue-light irradiation, achieving the modular construction of complex molecules. This strategy exhibits wide substrate scope; various oxime carbonates, imines, and structurally diverse alkenes can be smoothly converted, affording the target products in 21%–84% yields (Scheme 11). These products, serving as highly valuable synthetic hubs, can undergo downstream transformations such as selective hydrolysis or cyclization to rapidly construct complex heterocyclic frameworks like cyclic carbonates and 1,3-oxazepane derivatives, demonstrating immense potential in the construction of molecular diversity libraries. The successful scale-up to the gram scale further corroborates its practical synthetic value.

**SCHEME 11:**
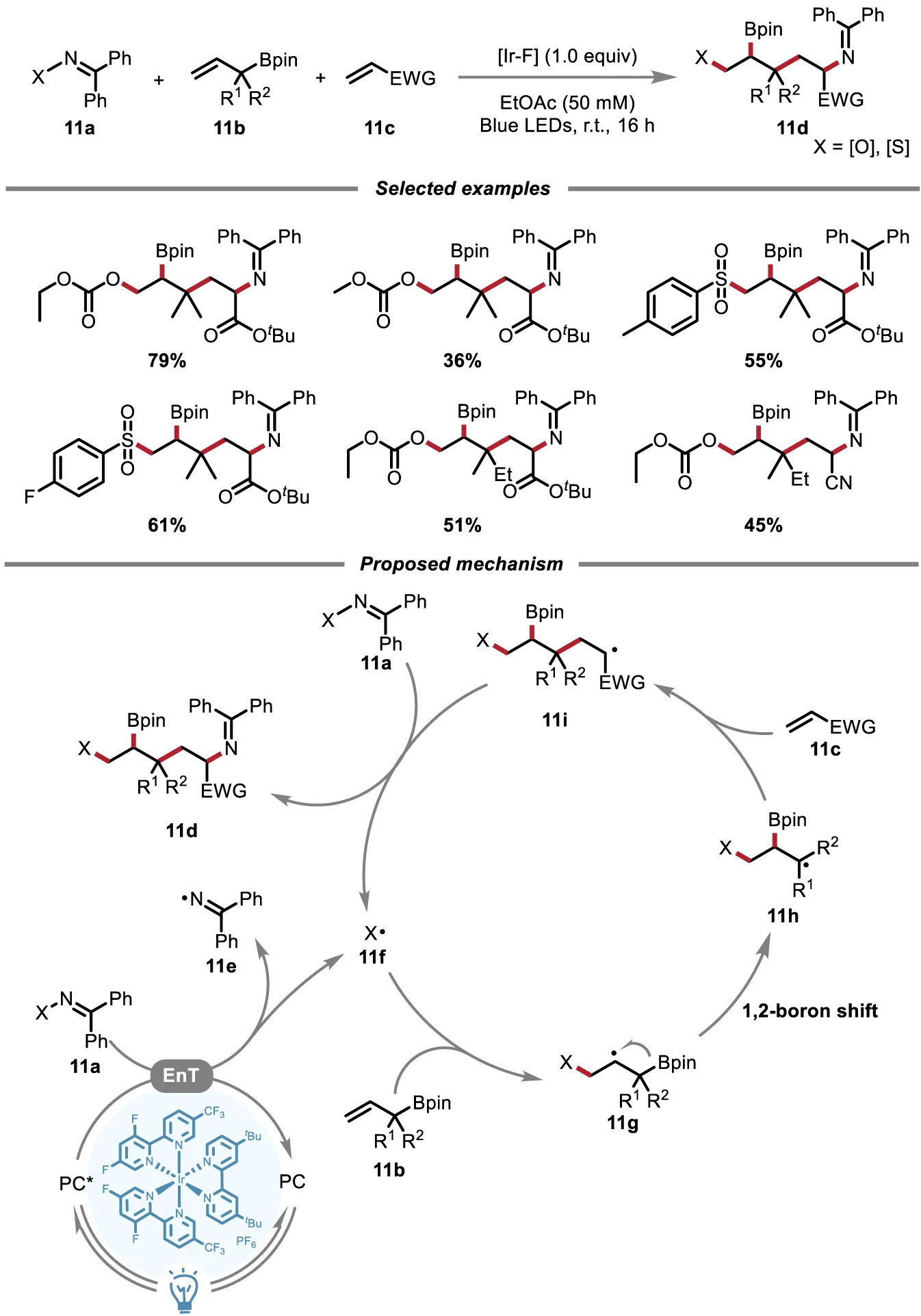
Three-component photochemical 1,2,5-trifunctionalization reaction.

Mechanistic studies indicate that the excited-state photocatalyst induces the homolytic cleavage of the N-X bond in the bifunctional reagent **11a** via triplet energy transfer, releasing a persistent amphiphilic iminyl radical **11e** and a transient electrophilic oxygen-centered radical **11f**. The latter undergoes a polarity-matched addition to the allylboronic ester **11b**, triggering a rapid 1,2-boron shift to generate a more stable nucleophilic tertiary carbon radical **11h**. This intermediate is subsequently captured by the electron-deficient Michael acceptor **11c**, generating the remote carbon radical **11i**. Ultimately, this intermediate **11i** undergoes a radical-radical cross-coupling with the persistent iminyl radical **11e**, or a radical chain reaction with the bifunctional reagent **11a**, to yield the 1,2,5-trifunctionalized target product **11d**. In the latter chain mechanism, the transient oxygen-centered radical **11f** is simultaneously released to maintain the reaction cycle.

### 3.5 C3 alternating copolymerization

In polymer chemistry, although the radical polymerization of C2 (vinyl) and C4 (diene) backbones has established a massive industrial foundation, the polymerization of C3 backbones has long been hindered due to the difficulty in stabilizing the β-carbon radical during chain propagation. To address this fundamental bottleneck, in 2026, the Pan group took an unconventional approach and developed a novel strategy that synergizes 1,2-boron shift with radical polarity matching, successfully achieving C3 alternating copolymerization (Xu et al., 2026). This system utilizes allylboronic esters and maleic anhydride as comonomers. Under conventional LPO thermal initiation, it operates efficiently to construct C3 backbone copolymers with strictly alternating sequences in 20%–91% yields (Scheme 12).

**SCHEME 12:**
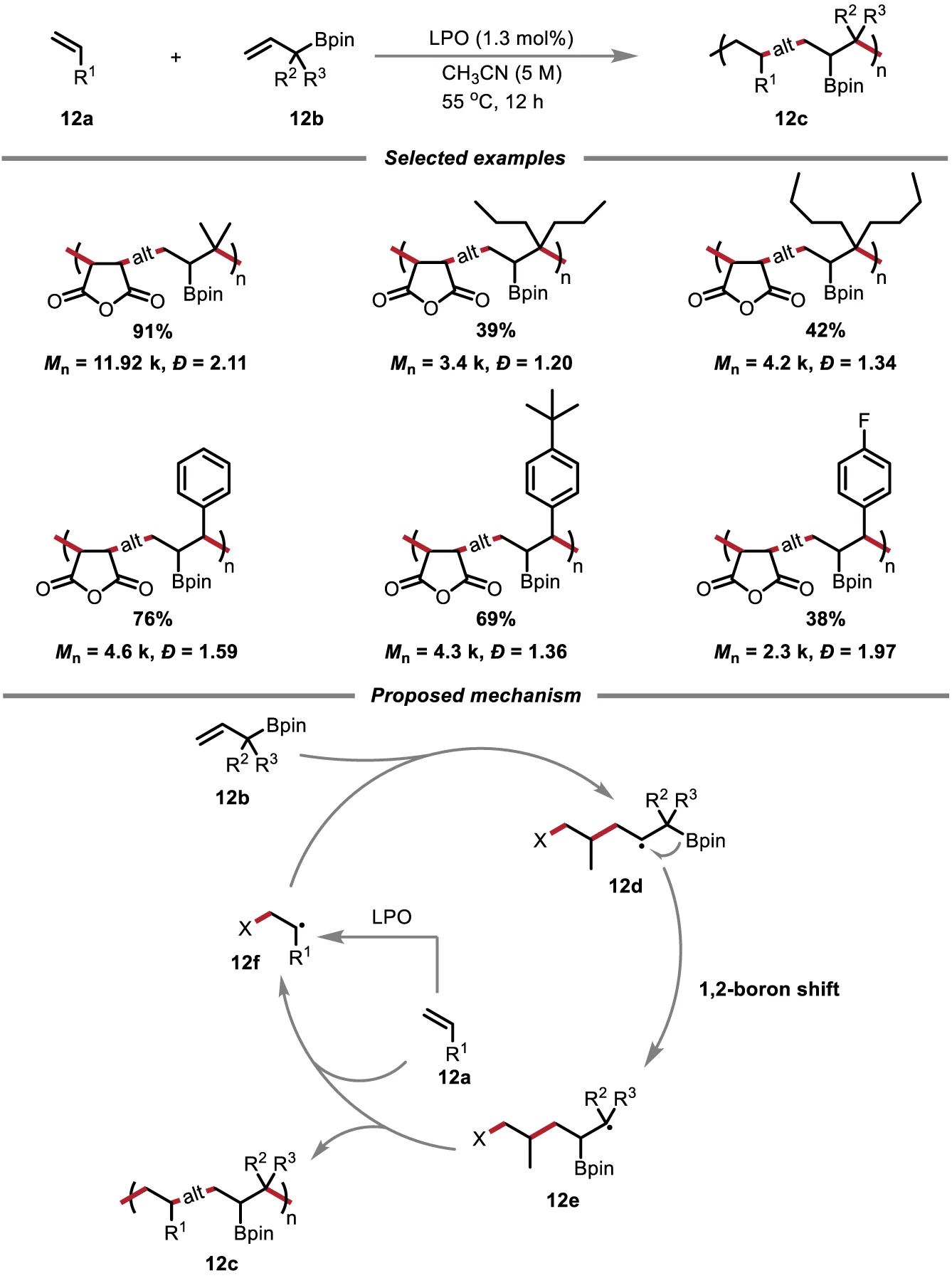
1,2-Boron shift and polarity matching-driven C3 alternating copolymerization.

Mechanistic studies indicate that in the presence of LPO, the maleic anhydride monomer **12a** generates an electrophilic radical **12f**. The secondary carbon radical **12d**, produced by the attack of the electrophilic radical **12f** on the allylboronic ester **12b**, is highly unstable and rapidly undergoes a 1,2-boron shift to yield the radical intermediate **12e**. Simultaneously, the polarity of the radical intermediate **12e** is inverted, becoming a nucleophilic tertiary carbon radical. Following the principle of polarity matching, this nucleophilic radical selectively adds to the highly electron-deficient maleic anhydride monomer **12a**, generating a new electrophilic propagating radical **12f**, which subsequently attacks the allylboronic ester **12b** again.

## 4 β-Boryl redox-active esters as precursors

### 4.1 β-Boryl NHPI esters

Although 1,2-boron shift strategies based on allylboronic esters have emerged, their reaction modes and synthetic potential remain far from being fully explored. How to break out of existing paradigms and develop novel activation strategies to expand the boundaries of the shift is the primary challenge currently facing this field. In particular, how to efficiently construct the crucial β-boryl radical intermediates in this process remains an urgent synthetic bottleneck to overcome, acting as the core pivot that determines whether this strategy can unleash its broad application potential.

To expand the 1,2-boron shift strategy from allylic substrates to a broader range of alkyl chain systems. In 2023, the Song group developed a novel strategy cascading photocatalytic decarboxylation with 1,2-boron shift, successfully achieving the diverse derivatization of organoboron molecules (Guo et al., 2023). This system utilizes β-boryl NHPI esters and various radical acceptors as substrates. Under the catalysis of a catalytic amount of Ir (ppy)_3_ and synergistic reduction by a Hantzsch ester (HE)/DIPEA system, it operates efficiently under blue-light irradiation at room temperature. This strategy exhibits broad substrate compatibility and transformation diversity. It is not only compatible with a broad range of β-boryl NHPI ester substrates, but by switching the radical acceptor, it can respectively achieve highly efficient decarboxylative alkynylation, allylation, and alkylation cross-couplings (Scheme 13). Particularly noteworthy is that this method can be seamlessly extended to β-*gem*-diboryl NHPI esters, paving a shortcut for the construction of high-value 1,2-diboron compounds. The successful demonstration on the gram scale and the rich derivatization of the products further highlight the strategic value of the resulting organoboron compounds as versatile synthetic handles.

**SCHEME 13:**
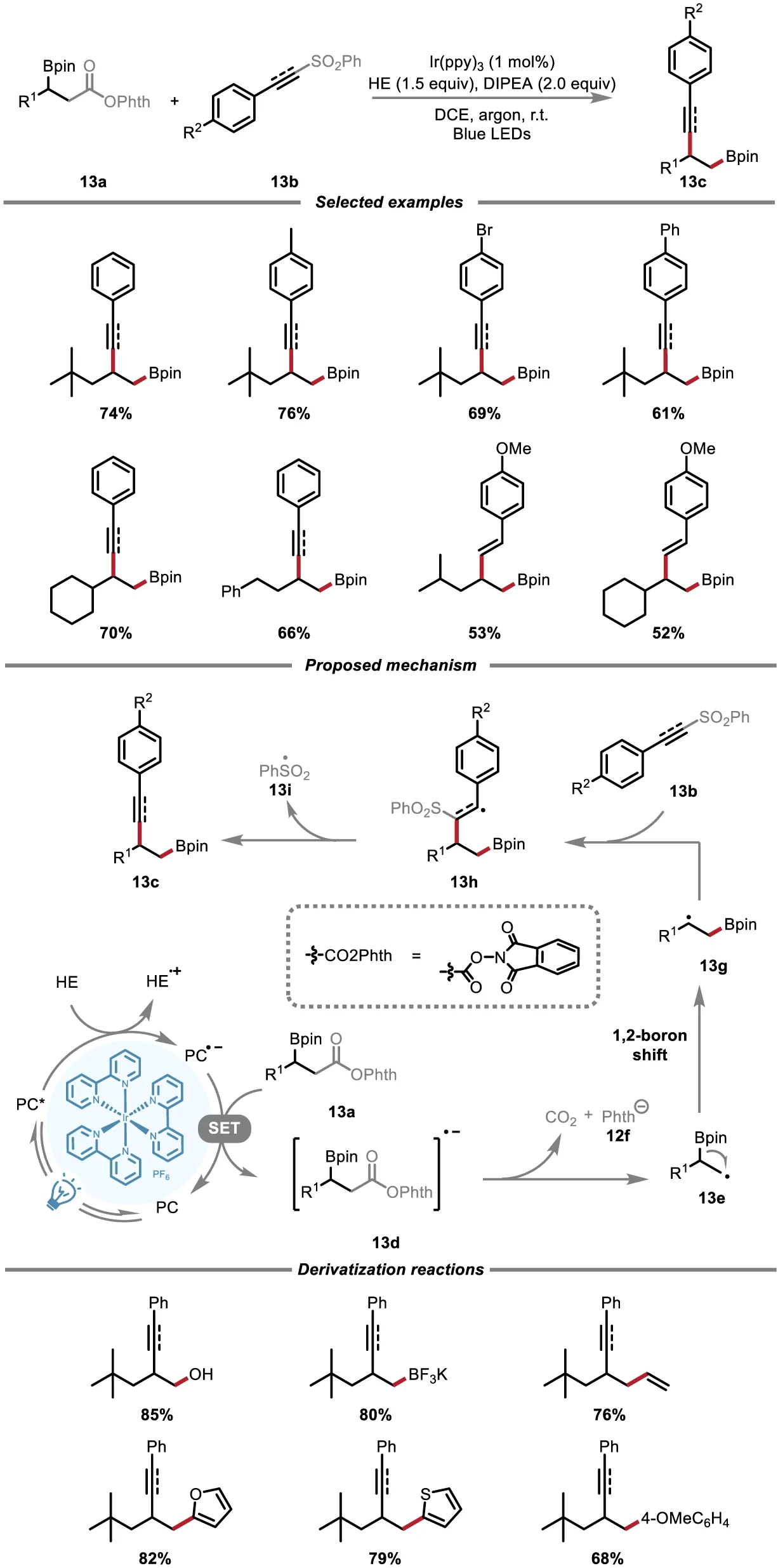
Photodecarboxylative 1,2-boron shift cross-coupling for the synthesis of organoboron compounds.

Mechanistic studies indicate that the light-excited reduced catalyst promotes the β-boryl NHPI esters **13a** to undergo SET and decarboxylation, releasing the β-boryl alkyl radical **13e**. Immediately thereafter, a thermodynamically driven 1,2-boron shift occurs, reshaping it into the more stable post-shift carbon radical **13g**. This carbon radical **13g** adds to an alkynyl aryl sulfone to yield the radical intermediate **13h**, which subsequently undergoes elimination of the sulfonyl group to afford the target product **13c**. Ultimately, the Hantzsch ester radical cation provides a proton, closing the cycle and regenerating the catalytic system.

The synergistic integration of N-heterocyclic carbene (NHC) catalysis and photoredox catalysis has provided a powerful tool for asymmetric synthesis, but its application in the functionalization of boron chemistry remains challenging. In 2024, the Li group developed a novel 1,2-boron shift acylation strategy driven by the synergy of NHC and photocatalysis, successfully achieving the highly efficient cross-coupling of aldehydes with β-boryl tetrachloro-N-hydroxyphthalimide (TCNHPI) esters (Huang et al., 2024). In the presence of Cs_2_CO_3_,through the synergistic action of an NHC precursor and an iridium photocatalyst, this system operates efficiently under blue-light irradiation and mild heating conditions, constructing a series of structurally diverse β-boryl ketones (Scheme 14). This strategy exhibits broad substrate adaptability and late-stage derivatization capabilities; various aromatic aldehydes, heterocyclic aldehydes, and even complex pharmaceutical molecules and natural product derivatives can be smoothly converted, with product yields reaching up to 84%. The successful gram-scale scale-up and the diverse derivatization transformations of the C-B bond further highlight the application potential of the resulting β-boryl ketones as valuable building-block scaffolds.

**SCHEME 14:**
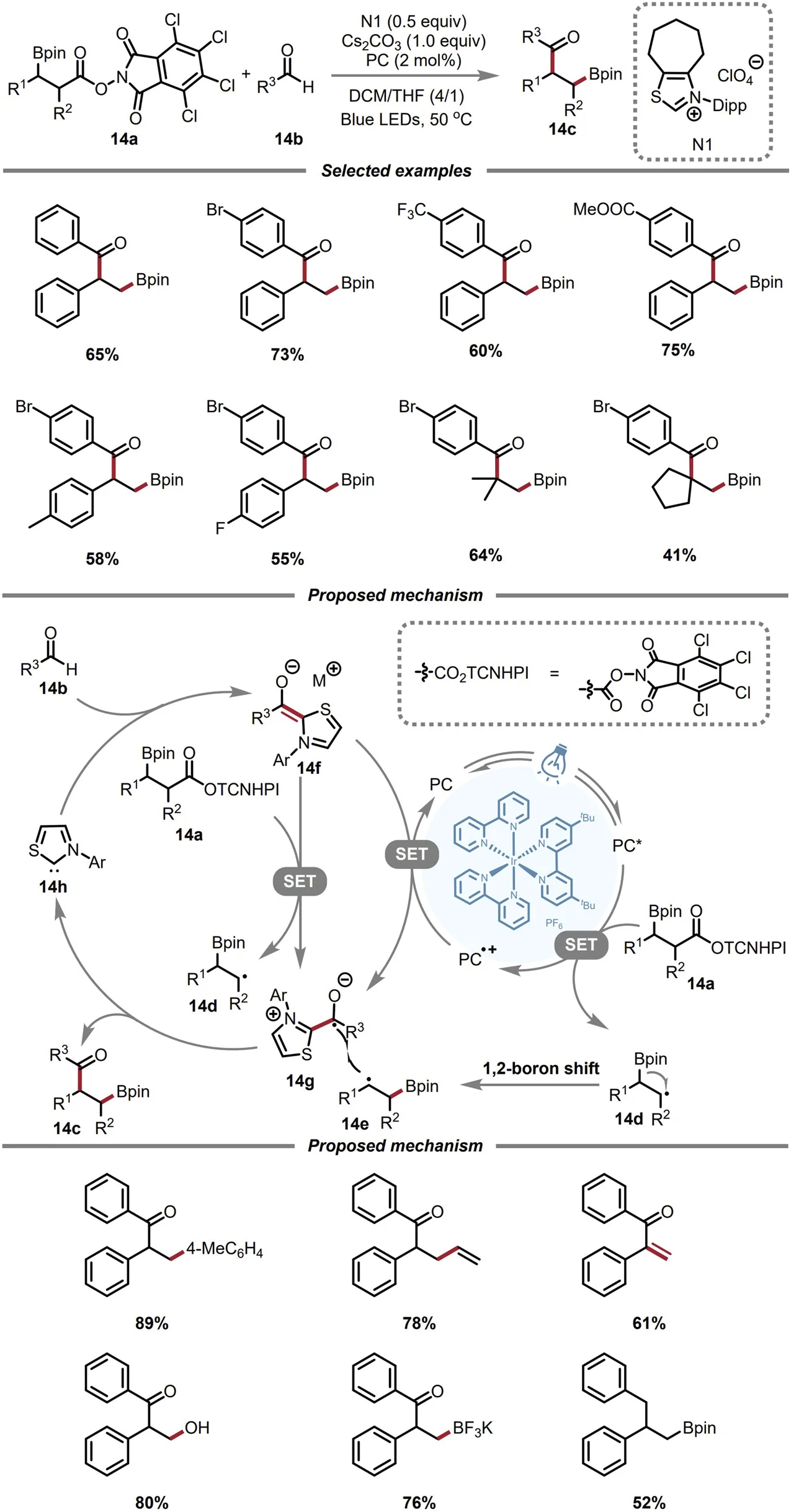
Photocatalysis accelerates N-heterocyclic carbene-catalyzed 1,2-boron shift acylation.

Mechanistic studies indicate that the light-excited iridium catalyst activates the β-boryl ester **14a** via SET. The released β-boryl alkyl radical **14d** rapidly undergoes a thermodynamically driven 1,2-boron shift, converting into the more stable post-shift radical **14e**. Meanwhile, the electron-rich Breslow intermediate **14f**, formed by the condensation of the aldehyde **14b** and the NHC catalyst **14h**, acts as a reducing agent to regenerate the photocatalyst and *in situ* generates the persistent acyl radical **14g**. Ultimately, these two radical species originating from distinct catalytic cycles undergo a highly efficient radical-radical cross-coupling, precisely constructing the target product **14c** while releasing the NHC catalyst to close the catalytic cycle.

Beyond the construction of carbon-carbon bonds, this strategy can equally be extended to heteroatom functionalization. In 2025, the Song group developed a novel visible-light-driven 1,2-boron shift thioetherification strategy, successfully achieving the highly efficient construction of β-thioalkylboronic esters (Lin et al., 2025). This system utilizes β-boryl NHPI esters and thiosulfonates as substrates. Under the catalysis of a catalytic amount of Ir (ppy)_3_ and synergistic reduction by DIPEA, the reaction operates efficiently under blue-light irradiation at room temperature, providing an entirely new pathway for the sulfur functionalization of organoboron molecules. This strategy exhibits broad substrate scope; various aryl and alkyl thiosulfonates, as well as structurally diverse β-boryl NHPI esters, can be smoothly converted to construct a series of target molecules in 51%–86% yields (Scheme 15). The successful scale-up to the gram scale and the rich downstream derivatization of the products further highlight the practical value of this synthetic building block in the derivatization of complex molecules.

**SCHEME 15:**
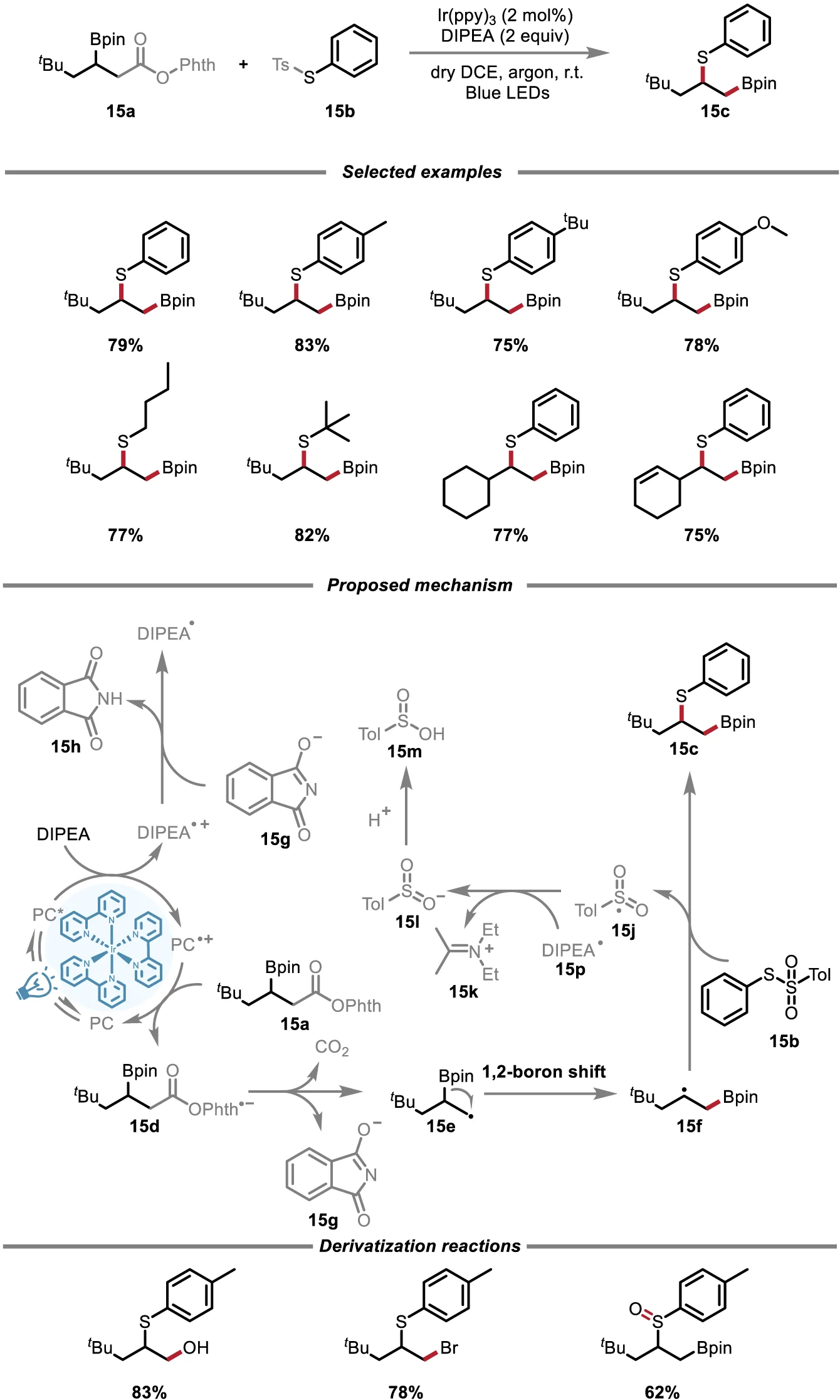
Photodecarboxylative 1,2-boron shift for the synthesis of β-thioalkylboronic esters.

Mechanistic studies indicate that the light-excited iridium catalyst undergoes reductive quenching by DIPEA to generate a strongly reducing Ir(II) species. This highly reducing Ir(II) species induces the decarboxylation of the NHPI ester **15a** via SET, releasing the β-boryl alkyl radical **15e**. This radical immediately undergoes a thermodynamically driven 1,2-boron shift, reshaping it into the more stable carbon-centered radical **15f**. Ultimately, this intermediate **15f** selectively attacks the S-S bond of the thiosulfonate **15b**, constructing a C-S bond while simultaneously releasing the benzenesulfonyl radical **15j**. The benzenesulfonyl radical **15j** then undergoes reduction and protonation to close the catalytic cycle and regenerate the system.

With the continuous expansion of photocatalytic systems, multicomponent shift reactions have also been realized. In 2026, the Wu group developed a novel visible-light-driven 1,2-boron shift carbonylation strategy, successfully achieving the highly efficient synthesis of β-boryl thioesters (Cui et al., 2026). Utilizing the organic dye Acid Red 94 as a catalyst, this system promotes the smooth conversion of β-boryl NHPI esters and thiosulfonates under mild light irradiation and a high-pressure CO atmosphere, constructing a series of structurally diverse target molecules in 34%–73% yields (Scheme 16). This strategy not only exhibits good substrate generality and demonstrated on gram scale potential, but the resulting β-boryl thioester products have also been proven to be high-value building blocks. They can be conveniently integrated into complex molecular scaffolds through downstream derivatizations such as oxidation, vinylation, and conversion to trifluoroborate salts.

**SCHEME 16:**
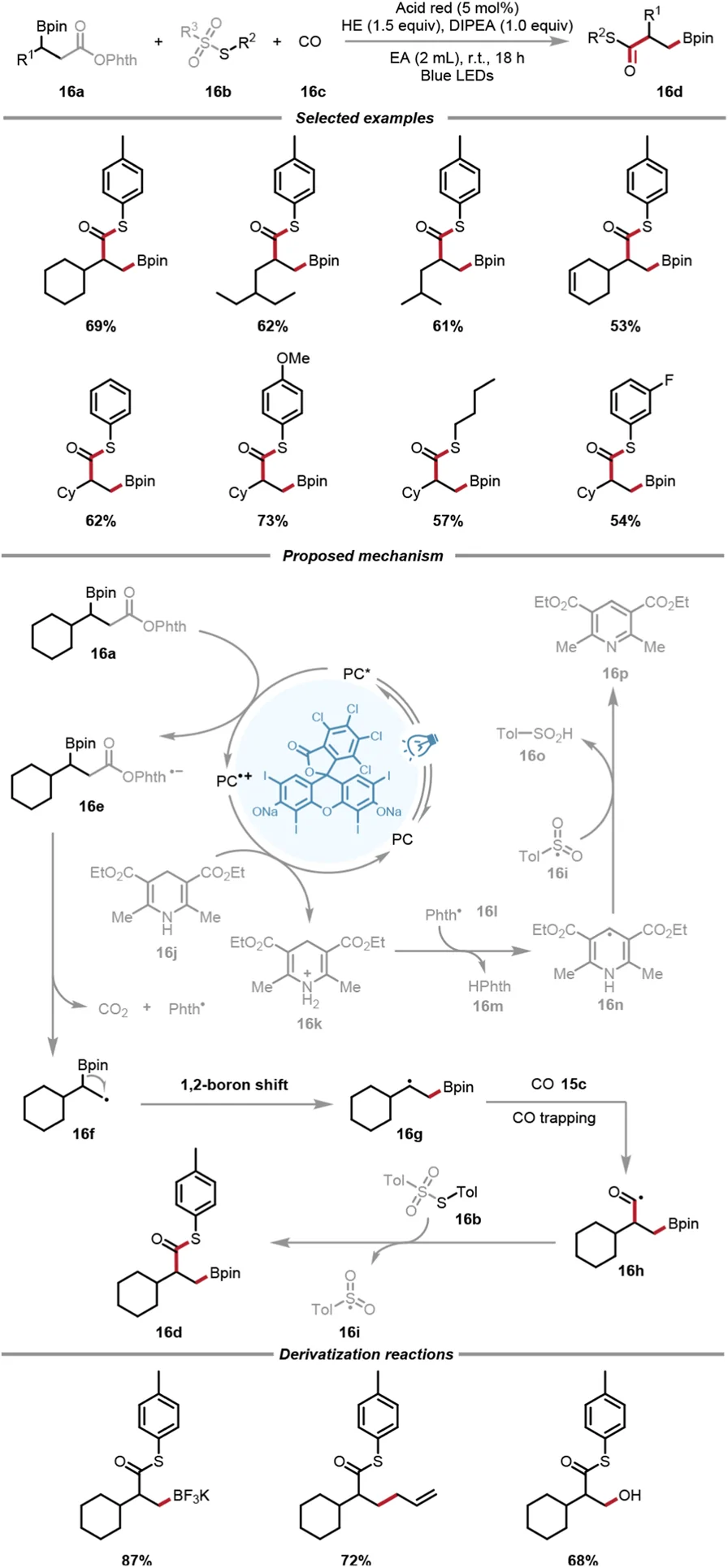
Photocatalytic 1,2-boron shift carbonylation for the synthesis of β-boryl thioesters.

Mechanistic studies indicate that the light-excited catalyst induces the NHPI ester **16a** to undergo SET and decarboxylation. The released β-boryl alkyl radical **16f** immediately undergoes a thermodynamically driven 1,2-boron shift, converting into the more stable carbon radical intermediate **16g**. This intermediate is captured by carbon monoxide to form the acyl radical **16h**, which ultimately undergoes a radical cross-coupling with the thiosulfonate **16b**, constructing a C-C bond while simultaneously closing the cycle to regenerate the catalytic system.

Notably, in addition to reliance on noble metal or photocatalytic systems, strategies mediated by inexpensive metals have also provided an important complement to this field. The Zhou group developed a zinc-mediated 1,2-boron shift and radical cascade strategy, successfully achieving the highly efficient construction of *gem*-difluoroalkenylboronic esters (Pang et al., 2025). Discarding the dependence on noble metals and photocatalysis, this system utilizes only inexpensive zinc powder as a mediator to drive the cross-coupling of β-boryl NHPI esters with α-trifluoromethyl alkenes under mild thermal conditions, constructing a series of structurally novel target molecules in 25%–97% yields (Scheme 17). The successful gram-scale scale-up and the rich downstream derivatizations of the products (such as vinylation, arylation, oxidation, and hydrogenation) highlight the high added value of these organoboron building blocks in complex molecule synthesis.

**SCHEME 17:**
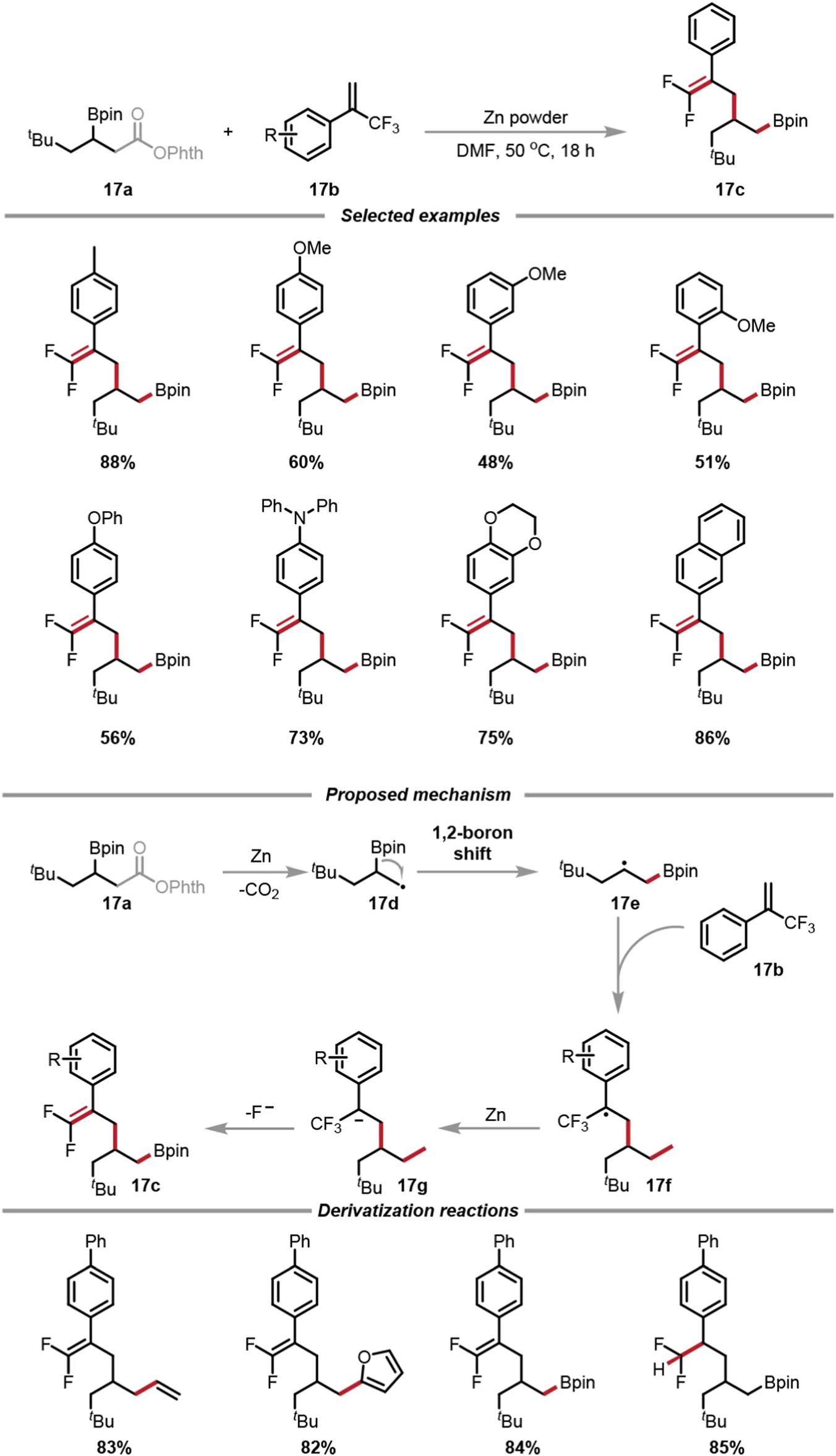
Zn-mediated 1,2-boron shift for the synthesis of *gem*-difluoroalkenylboronic esters.

Mechanistic studies indicate that zinc powder, acting as an electron donor, triggers the decarboxylation of the NHPI ester **17a**. The released radical **17d** rapidly undergoes a thermodynamically driven 1,2-boron shift, reshaping into the nucleophilic carbon-centered radical **17e**. Following the radical addition of this intermediate to the electron-deficient alkene **17b**, a subsequent single-electron reduction and a crucial β-fluoride elimination occur, precisely introducing the *gem*-difluoro moiety while constructing the C=C double bond to afford the final product **17c**.

### 4.2 β-Boryl oxime esters

In 2026, the Xia group reported a strategy based on photocatalytic energy transfer (EnT) for the modular synthesis of δ-amino boronates via radical 1,2-boron shift (Li S.-S. et al., 2026). β-boryl oxime esters were employed as bifunctional reagents, with Ir-F as the photocatalyst and acetonitrile as the solvent. The reaction was conducted at room temperature under 450 nm blue LED irradiation for 12 h under an inert atmosphere. This reaction system is compatible with various unsaturated systems including alkenes, C=N, N=N bonds, and alkyne-alkene relay systems, affording a series of target products in yields ranging from 22% to 95% (Scheme 18). A gram-scale experiment successfully produced the target compound in 70% isolated yield on a 2 mmol scale. Furthermore, the authors performed diverse late-stage derivatizations of the resulting δ-amino boronates: converting the boronate to a δ-amino alcohol via oxidation, selectively reducing the imine to an amine via photocatalytic reduction, synthesizing bioactive molecules through amide coupling with Ibuprofen, and preparing stable amine hydrochlorides via acidolysis.

**SCHEME 18:**
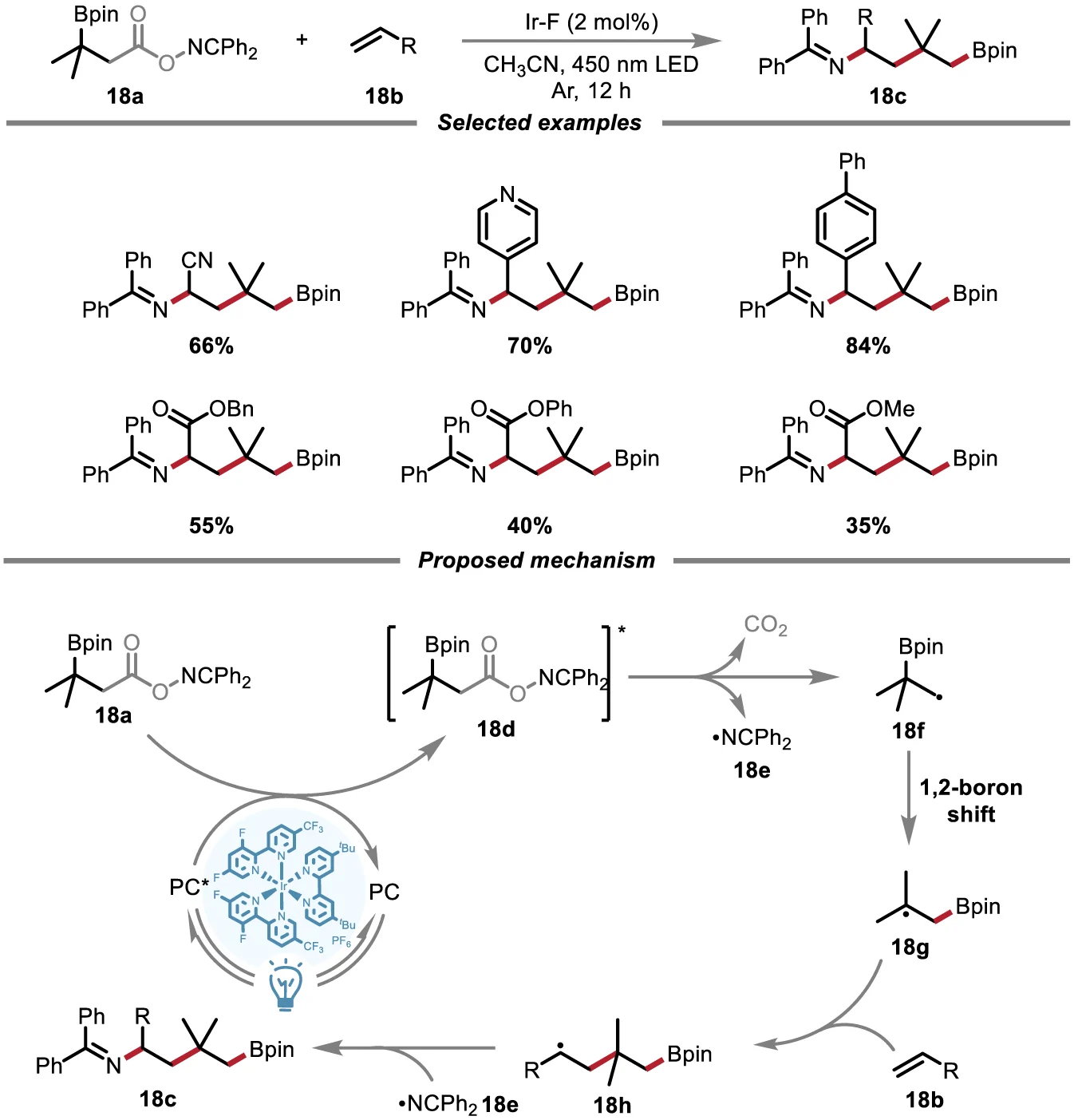
Photoinduced energy transfer-catalyzed radical 1,2-boron shift for the aminoboration of alkenes.

Mechanistic studies indicate that the Ir-F photocatalyst is promoted to a triplet excited state under 450 nm blue light irradiation. The excited photocatalyst transfers energy to substrate **18a** via EnT. The excited species **18d** undergoes decarboxylative cleavage, releasing carbon dioxide and generating carbon-centered radical **18f** and iminyl radical **18e**. Subsequently, the carbon-centered radical **18f** undergoes intramolecular 1,2-boron shift to form the distal β-boryl radical **18g**. Radical **18g** adds to the unsaturated acceptor **18b** to generate the adduct radical **18h**, which then undergoes radical-radical cross-coupling with the iminyl radical **18e** to afford the final product **18c**.

## 5 Alkyl boronic esters as precursors

Although radical-mediated 1,2-boron shift has proven to be an effective tool for constructing complex boronates, its application has long been restricted by a dependence on specific substrate structures. Previous strategies have inevitably increased the overall number of synthetic steps, failing to fundamentally achieve the ideal state of editing the position without altering the skeleton. Therefore, developing a general strategy capable of directly utilizing the ubiquitous, unactivated C(sp^3^)-H bonds inherent to ordinary alkyl boronates as the reaction starting point, without requiring any pre-functionalization, has become a core and urgent challenge in this field.

In 2026, addressing this critical limitation, the Xi group reported a catalytic 1,2-boron shift reaction (Zhang W. et al., 2026). By initially converting various functional groups into pinacol boronates, executing the boron shift, and subsequently transforming them back to the original functional groups, this approach achieved the formal positional editing of diverse functional groups, including alcohols, amines, carboxylic acids, alkenes, and bromides. This provides a general strategy for the rapid acquisition of regioisomers. The reaction utilizes [TBA][FeCl_4_] as the hydrogen atom abstraction catalyst and TRIP_2_S_2_ as the hydrogen atom donation catalyst, proceeding in acetonitrile under 390 nm light-emitting diode (LED) irradiation. This reaction system can be applied to a broad range of tertiary and secondary boronates, affording a series of products in 22%–85% yields (Scheme 19). Furthermore, this study not only achieved the late-stage editing of complex pharmaceutical molecules such as cholestanone, but also accomplished precise positional isomerization of functional groups. Meanwhile, by modulating the concentration of the hydrogen atom donation catalyst, the reaction directionality can be reversed, enabling different regioisomers to be selectively obtained as the major products. Additionally, this method enables a chain-walking remote shift through successive 1,2-boron shifts and can be utilized to override the inherent regioselectivity of classical organic reactions, thereby affording non-classical products.

**SCHEME 19:**
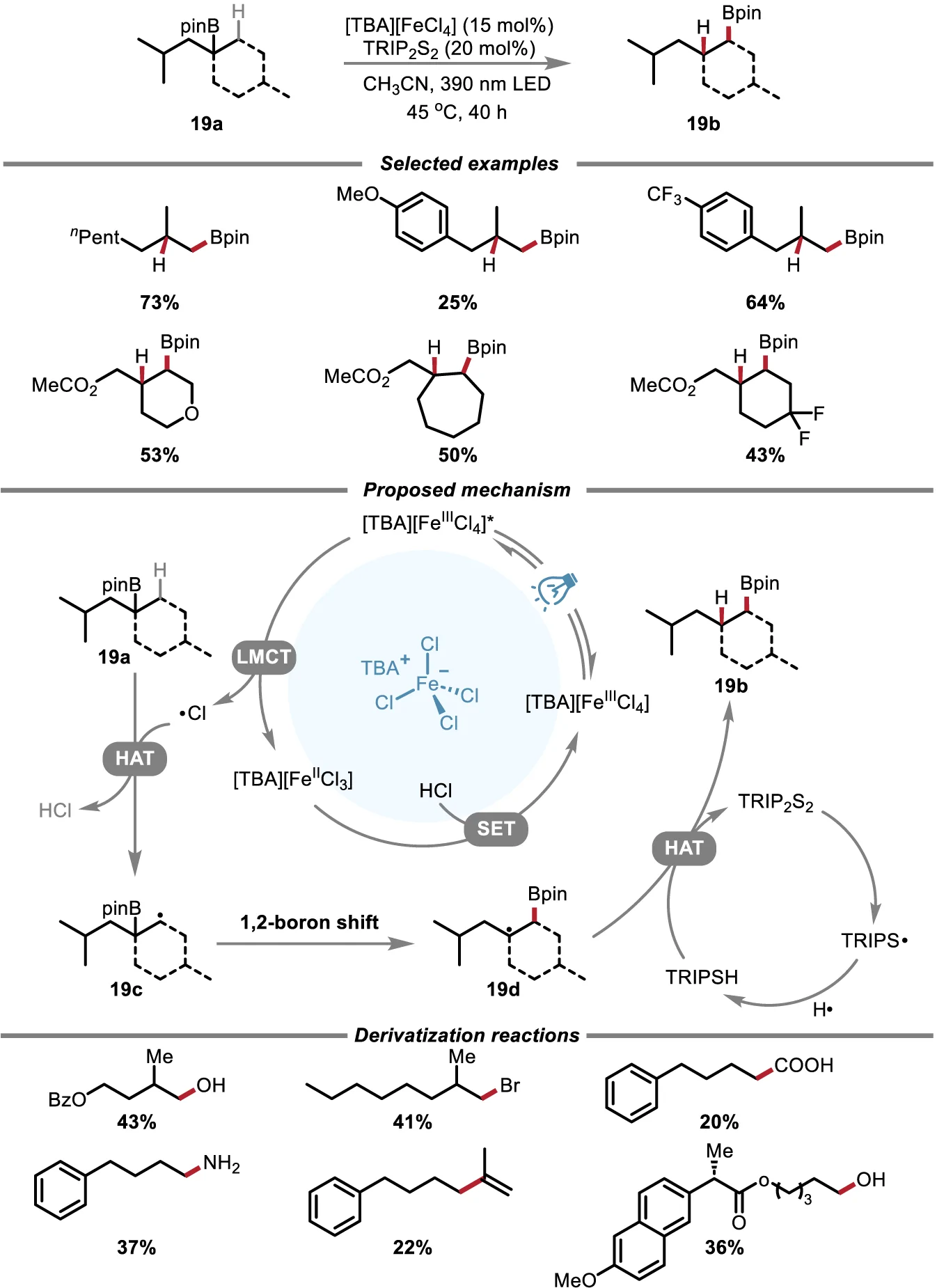
Positional editing of diverse functional groups through radical 1,2-boron shift.

Mechanistic studies reveal that under visible-light irradiation, tetrabutylammonium ferrate (TBAFeCl_4_) undergoes ligand-to-metal charge transfer (LMCT) to generate a chlorine radical. This species subsequently abstracts a hydrogen atom from the C–H bond of boronic ester **19a**, affording carbon-centered radical intermediate **19c**. Intermediate **19c** then rapidly engages in a 1,2-boron shift to furnish the rearranged carbon-centered radical **19d**. Finally, radical **19d** undergoes HAT from a thiophenol derivative to deliver the desired product **19b**.

Subsequently, in 2026, Deng’s team reported a novel method for the unactivated C(sp^3^)-H functionalization of alkyl boronates catalyzed by photoinduced LMCT via a 1,2-boron shift pathway (Li D. et al., 2026). Proceeding under mild conditions, this approach utilizes FeCl_3_ as the photocatalyst and HCl as an additive under 395 nm LED irradiation to achieve highly regioselective alkylation and arylation of alkyl boronates with a variety of electron-deficient alkenes and heteroaromatics. A series of products were obtained in yields ranging from 40% to 96% (Scheme 20). Furthermore, the authors demonstrated the synthetic utility of this approach by converting the alkyl boronates into high-value functionalized molecules such as alcohols, esters, and fluoroborates. This proved that this photocatalytic method is not limited to C-C bond construction, but can also be conveniently transformed into various synthetic intermediates, significantly expanding its potential applications in medicinal chemistry and materials science.

**SCHEME 20:**
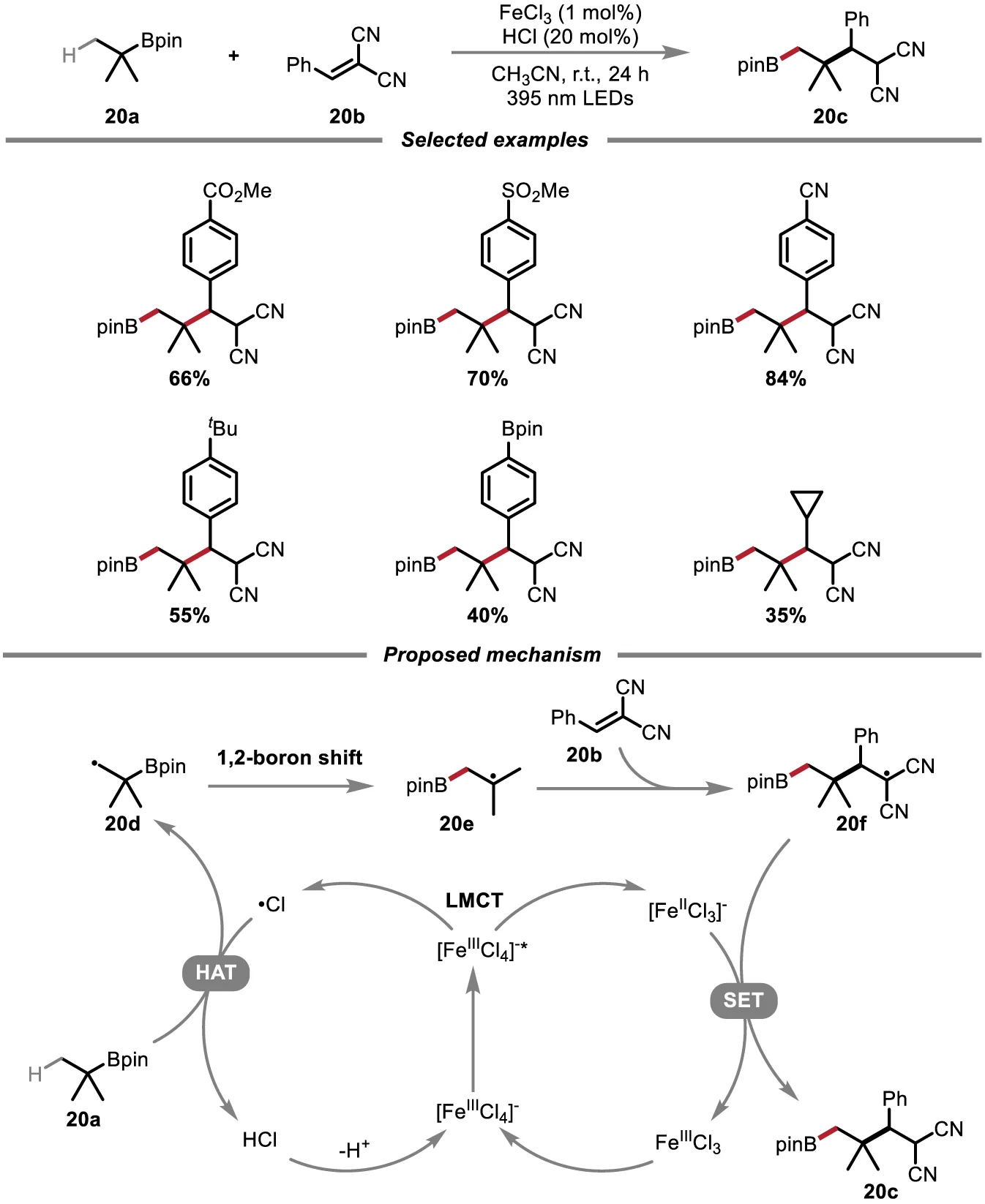
Photoinduced LMCT-catalyzed regioselective C(sp^3^)-H functionalization of boronates via a 1,2-boron shift pathway.

Mechanistic studies indicate that under light irradiation, [FeCl_4_]^-^ undergoes an LMCT to generate a chlorine radical. Subsequently, the chlorine radical abstracts a hydrogen atom from the terminal C(sp^3^)-H bond of **20a**, forming the primary alkyl radical intermediate **20d**. Intermediate **20d** rapidly undergoes a 1,2-boron shift to yield the more stable tertiary alkyl radical **20e**. The tertiary alkyl radical **20e** undergoes nucleophilic addition to the electron-deficient alkene **20b** to generate radical **20f**; **20f** then undergoes SET reduction followed by protonation to afford the product **20c**.

How to further eliminate the dependence on directing groups and stoichiometric reagents to achieve the most fundamental and native B-H position exchange has remained one of the significant challenges in this field. In 2026, the Xu group reported a photocatalytic method capable of realizing the direct 1,2-interchange of the boryl group and the hydrogen atom in alkyl boronic esters via a reversible C-H sampling strategy under directing-group-free and stoichiometric-reagent-free conditions (Xie et al., 2026). The reaction utilizes tetrabutylphosphonium decatungstate (TBPDT) as the photo-induced hydrogen atom abstraction (HAA) catalyst and 2,4,6-triisopropylbenzenethiol (T3) as the hydrogen atom donor (HAD) catalyst, proceeding in acetonitrile (MeCN) under 365 nm LED irradiation. The process is conducted at room temperature without the need for additional functional or directing groups. This methodology is broadly applicable to a wide range of tertiary and secondary alkyl boronic esters, including substrates containing linear, monocyclic, and bicyclic frameworks, as well as various functional groups, with yields ranging from 28% to 88% and excellent 1,2-regioselectivity (Scheme 21). Furthermore, this reaction system can be implemented in gram-scale flow synthesis and cascaded with other borylation reactions to construct boronic ester building blocks that are difficult to synthesize via traditional methods.

**SCHEME 21:**
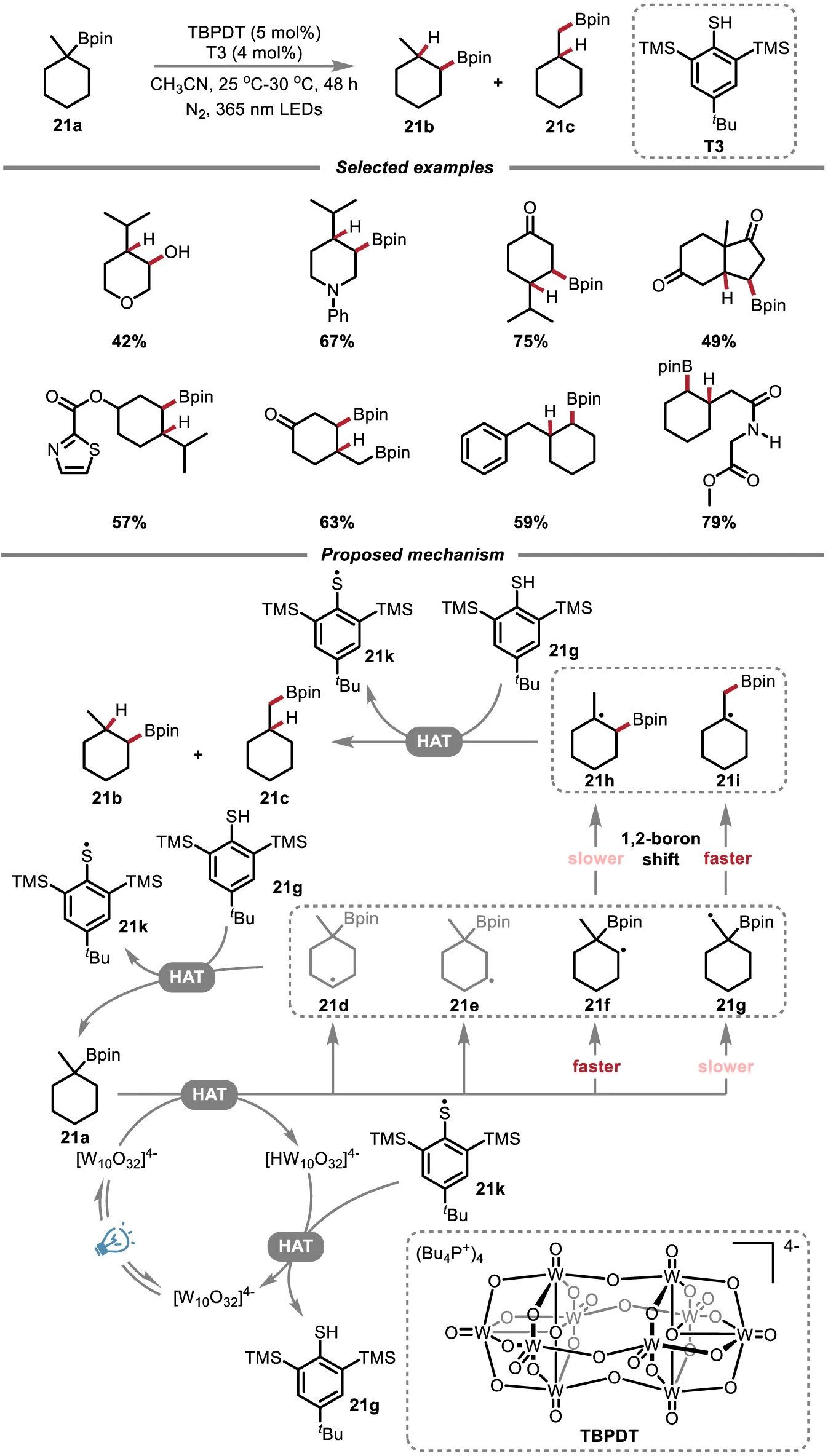
1,2-Boron/hydrogen transposition via reversible C-H sampling.

Mechanistic studies indicate that, first, the photocatalyst TBPDT is excited under light irradiation and non-selectively cleaves various C(sp^3^)-H bonds in the substrate via a HAT process. This generates different carbon radical intermediates **21d**, **21e**, **21f**, and **21g**, while TBPDT is simultaneously reduced to its reduced state. The thiophenol hydrogen atom donor catalyst then reacts with carbon radicals **21d** and **21e**, which have not undergone 1,2-boron shift, reverting them back to the original substrate state and enabling the reversible sampling of the C-H bonds.

When T3 is used as the HAD (hydrogen atom donor) reagent, the HAT rate is relatively slow due to its large steric hindrance, allowing a substantial amount of carbon radical **21f** to undergo 1,2-boron shift to yield radical intermediate **21h**. Conversely, when a less sterically hindered HAD reagent is used, the HAT rate is much faster. If carbon radicals **21f** and **21g** fail to migrate in time, they are rapidly hydrogenated back to the substrate by the HAD reagent. Therefore, the difference in the rate of 1,2-boron shift becomes the dominant factor determining the selectivity. Ultimately, the thiophenol catalyst transfers hydrogen atoms to **21h** and **21i** to form the final products **21b** and **21c**, while the thiophenol radical is regenerated via a HAT process with the reduced state of TBPDT.

## 6 Conclusion and outlook

Radical-mediated 1,2-boron shift has emerged as a transformative strategy for the construction of complex organoboron compounds. Broadly speaking, advances in this field have transitioned from classical strategies relying on pre-functionalized substrates and specific olefinic scaffolds to a more advanced stage capable of directly activating unactivated C(sp^3^)–H bonds for site editing.

Throughout this evolutionary process, the underlying principle has been the utilization of thermodynamically driven radical rearrangements, which effectively circumvent the regioselectivity limitations imposed by steric hindrance in traditional transition-metal catalysis. This not only provides a highly efficient pathway for the construction of sterically hindered secondary and tertiary alkylboronic sites, but by introducing concepts such as polarity matching and radical relay, it also achieves seamless compatibility with multicomponent cascade reactions and polymer copolymerization. Furthermore, the retention of the carbon–boron bond during the reaction provides a highly valuable synthetic handle for the late-stage modular derivatization of downstream complex molecules.

Despite these remarkable advances, the field still faces several pressing challenges before it can realize a more ideal and universally applicable paradigm for skeletal editing. First, the high reactivity and transient nature of radical intermediates render stereochemical control exceedingly difficult. There is an urgent need within the field to develop novel chiral ligands or bifunctional catalytic systems capable of engaging in effective asymmetric induction with radicals. This is crucial for overcoming the bottleneck of highly enantioselective shift and achieving the precise construction of chiral centers (Figure 3).

**FIGURE 3:**
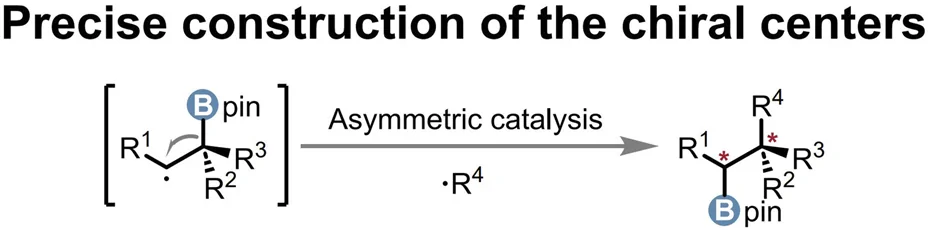
Future perspective.

Secondly, to substantially broaden the synthetic boundaries of this strategy, future methodological expansion must focus on two core directions. Horizontally, it is necessary to develop diverse types of pre-functionalized substrates and novel radical acceptors, thereby further enriching the structural diversity of the target scaffolds. Vertically, improving the catalytic efficiency and substrate scope of direct C(sp^3^)–H functionalization strategies remains a central challenge. Although recent emerging mechanisms, such as photoinduced LMCT, reversible C–H sampling, and cascade reaction-mediated chain walking, have shown preliminary success in the direct functionalization of unactivated C(sp^3^)–H bonds, elucidating and establishing a more precise and entirely predictable remote shift mode remains a critical hurdle for the future.

Finally, the introduction of ionic liquids or heterogeneous catalysts to enable catalyst recycling will undoubtedly establish a catalytic system that better aligns with the principles of green synthetic chemistry. In addition, it is noteworthy that electrocatalysis, as a green and sustainable strategy for radical generation, offers both precise potential control and compatibility with renewable energy sources. As an as-yet-unexplored activation mode, the electrocatalysis-driven radical 1,2-boron shift is expected to complement photocatalytic and thermocatalytic methods, thereby opening a sustainable new avenue for skeletal editing.

## References

[B1] Batey R. A. Smil D. V. (1999). The first boron-tethered radical cyclizations and intramolecular homolytic substitutions at boron. Angew. Chem. Int. Ed. 38 (12), 1798–1800. 10.1002/(SICI)1521-3773(19990614)38:12<1798::AID-ANIE1798>3.0.CO;2-0 29711210

[B2] Bonet A. Pubill-Ulldemolins C. Bo C. Gulyás H. Fernández E. (2011). Transition-metal-free diboration reaction by activation of diboron compounds with simple lewis bases. Angew. Chem. Int. Ed. 50 (31), 7158–7161. 10.1002/anie.201101941 21688368

[B3] Cui H. Qiao X. Shi T. Wei W. Yue H. Wang Z. (2025). Photoinduced EDA complex triggered difluoroalkylation of quinoxalin-2(1H)-ones with unactivated alkenes and fluoroalkyl bromides. Chem. Commun. 61 (91), 17890–17893. 10.1039/d5cc04758g 41114700

[B4] Cui X.-F. Wang L.-C. Wu X.-F. (2026). Photoredox borocarbonylation through 1,2-boron migration. Catal. Sci. Technol. 16 (1), 293–301. 10.1039/D5CY01255D

[B5] Das P. Linert W. (2016). Schiff base-derived homogeneous and heterogeneous palladium catalysts for the Suzuki–Miyaura reaction. Coord. Chem. Rev. 311, 1–23. 10.1016/j.ccr.2015.11.010

[B6] Dembitsky V. M. Al Quntar A. A. A. Srebnik M. (2011). Natural and synthetic small boron-containing molecules as potential inhibitors of bacterial and fungal quorum sensing. Chem. Rev. 111 (1), 209–237. 10.1021/cr100093b 21171664

[B7] Fyfe J. W. B. Watson A. J. B. (2017). Recent developments in organoboron chemistry: old dogs, new tricks. Chem 3 (1), 31–55. 10.1016/j.chempr.2017.05.008

[B8] Guo Y. Wang X. Li C. Su J. Xu J. Song Q. (2023). Decarboxylation of β-boryl NHPI esters enables radical 1,2-boron shift for the assembly of versatile organoborons. Nat. Commun. 14 (1), 5693. 10.1038/s41467-023-41254-1 PMC10502150 37709736

[B9] Hu P. Kazemi M. (2024). A comprehensive review on palladium nanocatalysts in carbonylative Suzuki-coupling reactions. J. Coord. Chem. 77 (12-14), 1324–1348. 10.1080/00958972.2024.2378041

[B10] Hu D. Li Y. Li R. Wang M. Zhou K. He C. (2024). Recent advances in reactive oxygen species (ROS)-responsive drug delivery systems for photodynamic therapy of cancer. Acta Pharm. Sin. B 14 (12), 5106–5131. 10.1016/j.apsb.2024.10.015 PMC11725102 39807318

[B11] Huang H. Yu Z.-Y. Han L.-Y. Wu Y.-Q. Jiang L. Li Q.-Z. (2024). *N*-Heterocyclic carbene catalytic 1,2-boron migrative acylation accelerated by photocatalysis. Sci. Adv. 10(30)**,** eadn8401. 10.1126/sciadv.adn8401 PMC11268412 39047096

[B12] Jana K. Studer A. (2022). Allylboronic esters as acceptors in radical addition, boron 1,2-Migration, and trapping cascades. Org. Lett. 24 (4), 1100–1104. 10.1021/acs.orglett.2c00039 PMC8822490 35080407

[B13] Jana K. Bhunia A. Studer A. (2020). Radical 1,3-Difunctionalization of allylboronic esters with concomitant 1,2-Boron shift. Chem 6 (2), 512–522. 10.1016/j.chempr.2019.12.022

[B14] Jiang X.-M. Liu X.-R. Chen A. Zou X.-Z. Ge J.-F. Gao D.-W. (2022). 1,2-Boryl migration enables efficient access to versatile functionalized boronates. Eur. J. Org. Chem. 2022 (9), e202101463. 10.1002/ejoc.202101463

[B15] Kaiser D. Noble A. Fasano V. Aggarwal V. K. (2019). 1,2-Boron shifts of β-Boryl radicals generated from bis-boronic esters using photoredox catalysis. J. Am. Chem. Soc. 141 (36), 14104–14109. 10.1021/jacs.9b07564 31461622 PMC7610657

[B16] Kong D. Zhang M. Zhang Y. Yu Z. Cao H. Wu J. (2023). Photocatalyzed regioselective hydrosilylation for the divergent synthesis of geminal and vicinal borosilanes. Nat. Commun. 14 (1), 2525. 10.1038/s41467-023-38224-y PMC10154379 37130840

[B17] Kumar K. N. Sahu C. Mahindrakar P. Dhaked D. K. Swain S. P. (2025). UV light-mediated generation of ketyl radicals with a concurrent 1,2-Boron shift and one-pot synthesis of benzoxaboroles under continuous-flow conditions. Org. Lett. 28 (1), 26–31. 10.1021/acs.orglett.5c04213 41436248

[B18] Lennox A. J. J. Lloyd-Jones G. C. (2014). Selection of boron reagents for Suzuki–Miyaura coupling. Chem. Soc. Rev. 43 (1), 412–443. 10.1039/C3CS60197H 24091429

[B19] Li J. Guo R. Li D. Yang J. Zhang Y. Gao H. (2025). Bortezomib enhances the efficacy of BCMA CAR-T therapy through up-regulating BCMA expression in myeloma cells. Int. Immunopharmacol. 148, 114113. 10.1016/j.intimp.2025.114113 39862629

[B20] Li D. Du Z. He W. Xu Y. Xue F. Deng H. (2026a). Photoinduced LMCT-catalyzed regioselective C(sp3)–H functionalization of boronates via a 1,2-Boryl migration pathway. Org. Lett. 28, 6054–6059. 10.1021/acs.orglett.6c01326 42077137

[B21] Li S.-S. Jiang Y.-S. Chen D.-N. Zhang F.-Q. Lei Y. Tang H.-T. (2026b). Harnessing energy-transfer catalysis for modular δ-aminoboronate synthesis via radical 1,2-boryl migration. Sci. China Chem. 69, 4457–4463. 10.1007/s11426-025-3285-0

[B22] Lin Y. Guo Y. Mu S. Lin G. Kong W. Lei X. (2025). Radical 1,2-Boron shift-enabled synthesis of β-Thioalkylboronic esters from β-Boryl NHPI esters. Org. Lett. 27 (35), 9825–9830. 10.1021/acs.orglett.5c03295 40853835

[B23] Liu L. Dong Y. Pang B. Ma J. (2014a). [Bmim]PF6-Promoted ligandless Suzuki–Miyaura coupling reaction of potassium aryltrifluoroborates in water. J. Org. Chem. 79 (15), 7193–7198. 10.1021/jo500840s 24979337

[B24] Liu L. Dong Y. Tang N. (2014b). A highly efficient and recyclable ligand-free protocol for the suzuki coupling reaction of potassium aryltrifluoroborates in water. Green Chem. 16 (4), 2185–2189. 10.1039/C3GC42182A

[B25] Liu L. Wang W. Xiao C. (2014c). A simple and efficient protocol for Suzuki coupling reactions of aryl chlorides and aryl bromides in aqueous DMF. J. Organomet. Chem. 749, 83–87. 10.1016/j.jorganchem.2013.09.026

[B26] Liu L. Liu M. Yang J. Zhu B. Wang X. (2018). An efficient ligandless palladium-catalyzed protocol for suzuki-miyaura coupling reaction of potassium aryltrifluoroborates in water. Curr. Org. Chem. 22 (20), 2023–2028. 10.2174/1385272822666180913110957

[B27] Liu L. Zhao K. Li W. Liu M. Chen Y. Dong Y. (2019). Synthesis of heterobiaryls via Suzuki-Miyaura coupling reaction of potassium aryltrifluoroborates with heteroaryl halides in aqueous systems. Appl. Organomet. Chem. 33 (4), e4831. 10.1002/aoc.4831

[B28] Liu J. Lu L. Wood D. Lin S. (2020). New redox strategies in organic synthesis by means of electrochemistry and photochemistry. ACS Cent. Sci. 6 (8), 1317–1340. 10.1021/acscentsci.0c00549 32875074 PMC7453421

[B29] Lu Z. Long Y. Wang Y. Wang X. Xia C. Li M. (2021). Phenylboronic acid modified nanoparticles simultaneously target pancreatic cancer and its metastasis and alleviate immunosuppression. Eur. J. Pharm. Biopharm. 165, 164–173. 10.1016/j.ejpb.2021.05.014 34020022

[B30] McCallum T. (2023). Emerging radical rearrangement reactions: the 1,2-boron shift. Green Synth. Catal. 4 (1), 10–19. 10.1016/j.gresc.2022.10.013

[B31] Messner K. Vuong B. Tranmer G. K. (2022). The boron advantage: the evolution and diversification of boron’s applications in medicinal chemistry. Pharmaceuticals 15 (3), 264. 10.3390/ph15030264 PMC8948683 35337063

[B32] Miyaura N. Suzuki A. (1995). Palladium-catalyzed cross-coupling reactions of organoboron compounds. Chem. Rev. 95 (7), 2457–2483. 10.1021/cr00039a007

[B33] Pang W.-J. Wang X.-C. Sun J. Li X.-S. Zhou M.-D. (2025). Zn-Mediated fragmentation of β-Boryl NHPI esters enabling the radical 1,2-Boron shift for the synthesis of gem-Difluoroalkenyl boronates. Org. Lett. 27 (20), 5270–5274. 10.1021/acs.orglett.5c01486 40369706

[B34] Paulus F. Stein C. Heusel C. Stoffels T. J. Daniliuc C. G. Glorius F. (2023). Three-component photochemical 1,2,5-Trifunctionalizations of alkenes toward densely functionalized lynchpins. J. Am. Chem. Soc. 145 (43), 23814–23823. 10.1021/jacs.3c08898 37852246

[B35] Qiao L. Zhang Z. Wu Y. Sun K. Li X. Chen X. (2026). Visible light-induced N-Phenylbenzo[c]phenothiazine-Catalyzed α-C(sp3)–H phosphonylation of secondary amines via intramolecular 1,5-HAT. Org. Lett. 28 (28), 9077–9082. 10.1021/acs.orglett.6c02556 42396727

[B36] Shen H.-C. Sandvoß A. Hughes W. B. Tyler J. L. McCormick McPherson L. J. Coles S. J. (2026). Electrophilic amination-induced 1,2-boronate migration for the modular synthesis of β-amino boronic esters. Nat. Chem. 10.1038/s41557-026-02206-6 PMC13623650 42443629

[B37] Smoum R. Rubinstein A. Dembitsky V. M. Srebnik M. (2012). Boron containing compounds as protease inhibitors. Chem. Rev. 112 (7), 4156–4220. 10.1021/cr608202m 22519511

[B38] Song S. Gao P. Sun L. Kang D. Kongsted J. Poongavanam V. (2021). Recent developments in the medicinal chemistry of single boron atom-containing compounds. Acta Pharm. Sin. B 11 (10), 3035–3059. 10.1016/j.apsb.2021.01.010 PMC8546671 34729302

[B39] Song H.-K. Yang H.-B. Jing J. Li H.-C. Sun K. Yang Z. (2026). Recyclable Cs2AgBiBr6 for photocatalytic synthesis of cyanomethylated oxindoles. J. Org. Chem. 91 (20), 6840–6849. 10.1021/acs.joc.6c00085 42090592

[B40] Sun K. Lv Q. Chen X. Qu L. Yu B. (2021). Recent advances in visible-light-mediated organic transformations in water. Green Chem. 23 (1), 232–248. 10.1039/D0GC03447A

[B41] Sun K. Ge C. Chen X. Yu B. Qu L. Yu B. (2024). Energy-transfer-enabled photocatalytic transformations of aryl thianthrenium salts. Nat. Commun. 15 (1), 9693. 10.1038/s41467-024-54079-3 PMC11549398 39516492

[B42] Tao X. Ni S. Kong L. Wang Y. Pan Y. (2022). Radical boron migration of allylboronic esters. Chem. Sci. 13 (7), 1946–1950. 10.1039/d1sc06760e PMC8848984 35308850

[B43] Wang J. Liu T. (2022). NF-κB signaling pathway and efficacy of bortezomib-based combination chemotherapy in patients with Non-Hodgkin’s lymphoma. Int. J. Pharmacol. 18 (5), 850–855. 10.3923/ijp.2022.850.855

[B44] Wang H. Han W. Noble A. Aggarwal V. K. (2022a). Dual Nickel/Photoredox‐Catalyzed site‐selective cross‐coupling of 1,2‐Bis‐Boronic esters enabled by 1,2‐Boron shifts. Angew. Chem. Int. Ed. 61 (34), e202207988. 10.1002/anie.202207988 PMC9543306 35779000

[B45] Wang H. Wu J. Noble A. Aggarwal V. K. (2022b). Selective coupling of 1,2‐bis‐boronic esters at the more substituted site through visible‐light activation of electron donor–acceptor complexes. Angew. Chem. Int. Ed. 61 (18), e202202061. 10.1002/anie.202202061 PMC9314813 35213775

[B46] Wang L. He S. Liu R. Xue Y. Quan Y. Shi R. (2024). A pH/ROS dual-responsive system for effective chemoimmunotherapy against melanoma via remodeling tumor immune microenvironment. Acta Pharm. Sin. B 14 (5), 2263–2280. 10.1016/j.apsb.2023.12.001 PMC11119573 38799639

[B47] Wang H.-S. Li H.-C. Yuan X.-Y. Sun K. Chen X.-L. Qu L. (2025). A hydrogen atom transfer-enabled photocatalytic system for direct heteroarylation of C(sp3)–H and C(sp2)–H bonds. Green Chem. 27 (17), 4655–4663. 10.1039/D4GC06209D

[B48] Wang D. Liu X. Zhang J. Zhao J. Li P. (2026). Photocatalytic [4 + 1] annulation of vinyl aziridines with alkyl halides. Org. Lett. 28 (13), 4201–4207. 10.1021/acs.orglett.6c00743 41870005

[B49] Xiang P. Sun K. Shi A. An J. Chen X. Qu L. (2025). Visible-light-induced 1,3-difunctionalization of allylboronic esters enabled by a 1,2-boron shift. Green Chem. 27 (6), 1820–1827. 10.1039/D4GC06330A

[B50] Xie L. Luo P. Lu T. Zheng W. Su Y. Wang J. (2026). 1,2-Boryl/hydrogen transposition via reversible C–H sampling. Chem 12, 102961. 10.1016/j.chempr.2026.102961

[B51] Xiong L. Chen S. Li S. He D. Wang Y. Zhang Q. (2025). ATP-responsive tumor targeted lipid nanoparticle for enhanced siRNA delivery and improved treatment efficacy in melanoma. J. Control. Release 382, 113622. 10.1016/j.jconrel.2025.113622 40120691

[B52] Xu P. Duan X. (2019). Recent progress in the suzuki-miyaura cross-coupling reactions in water. Chin. J. Org. Chem. 39 (12), 3315–3327. 10.6023/cjoc201908020

[B53] Xu L. Zhang S. Li P. (2015). Boron-selective reactions as powerful tools for modular synthesis of diverse complex molecules. Chem. Soc. Rev. 44 (24), 8848–8858. 10.1039/C5CS00338E 26393673

[B54] Xu X. Shang H. Zhang T. Shu P. Liu Y. Xie J. (2018). A stimuli-responsive insulin delivery system based on reversible phenylboronate modified cyclodextrin with glucose triggered host-guest interaction. Int. J. Pharm. 548 (1), 649–658. 10.1016/j.ijpharm.2018.07.020 29981896

[B55] Xu C. Kong Y. Kroeger A. A. Coote M. L. Pan X. (2026). Radical C3 alternating polymerization driven by 1,2-boryl migration via radical polarity matching. Nat. Synth. 5, 948–957. 10.1038/s44160-026-01005-8

[B56] Yang W. Zhang Z. Yang Z. Chen X. Qiao L. Chen X. (2025). Synergistic photocatalysis of acridinium salts and pyridine N-oxides for α-aminoalkylation with N-Methylsulfonamides. J. Org. Chem. 90 (46), 16403–16413. 10.1021/acs.joc.5c01813 41217425

[B57] Yang Z. Yue H. Ma C. Jian Y. Wei W. Yi D. (2026). Visible-light-induced Oximo-Thioesterification of alkenes with tert-Butyl nitrite and thioacids leading to α-Thioester oximes. J. Org. Chem. 91 (12), 4460–4468. 10.1021/acs.joc.6c00202 41834660

[B58] Zeng Y.-Y. Jiang J. Wen Y.-C. Zhuang C.-L. Ou L.-J. Yang Z. (2026). Decatungstate-photocatalyzed heterogeneous direct benzylation of N-heterocycles with benzaldehydes. Chin. Chem. Lett. 37 (4), 111776. 10.1016/j.cclet.2025.111776

[B59] Zhang W. Mao S. Peng M. Xi Y. (2026). Positional editing of diverse functional groups through radical 1,2-boron shifts. Nat. Synth. 5, 1021–1031. 10.1038/s44160-026-01018-3

[B60] Zhang Z. Qiao L. Sun K. Li X. Jiang Z. Chen X. (2026). When dowd–beckwith reaction meets photocatalysis. Org. Biomol. Chem. 24 (28), 5819–5829. 10.1039/d6ob00896h 42429414

